# A drug screening to identify novel combinatorial strategies for boosting cancer immunotherapy efficacy

**DOI:** 10.1186/s12967-023-03875-4

**Published:** 2023-01-13

**Authors:** Zongliang Zhang, Guoqing Wang, Kunhong Zhong, Yongdong Chen, Nian Yang, Qizhong Lu, Boyang Yuan, Zeng Wang, Hexian Li, Liping Guo, Ruyuan Zhang, Zhiguo Wu, Meijun Zheng, Shasha Zhao, Xin Tang, Bin Shao, Aiping Tong

**Affiliations:** 1 State Key Laboratory of Biotherapy and Cancer Center, Research Unit of Gene and Immunotherapy, Chinese Academy of Medical Sciences, Collaborative Innovation Center of Biotherapy, West China Hospital, Sichuan University, Chengdu, 610041 Sichuan Province China; 2 Department of Neurosurgery, West China Hospital, West China Medical School, Sichuan University, Chengdu, 610041 Sichuan Province China; 3 Department of Otolaryngology, Head and Neck Surgery, West China Hospital, West China Medical School, Sichuan University, Chengdu, 610041 Sichuan Province China; 4 State Key Laboratory of Oral Diseases, National Clinical Research Center for Oral Diseases, West China Hospital of Stomatology, Sichuan University, Chengdu, 610041 China

**Keywords:** Small molecular screening, JK184, B7-H3 CAR T, ICBs, Solid tumor

## Abstract

**Background:**

Chimeric antigen receptor (CAR) T cells and immune checkpoint blockades (ICBs) have made remarkable breakthroughs in cancer treatment, but the efficacy is still limited for solid tumors due to tumor antigen heterogeneity and the tumor immune microenvironment. The restrained treatment efficacy prompted us to seek new potential therapeutic methods.

**Methods:**

In this study, we conducted a small molecule compound library screen in a human BC cell line to identify whether certain drugs contribute to CAR T cell killing. Signaling pathways of tumor cells and T cells affected by the screened drugs were predicted via RNA sequencing. Among them, the antitumor activities of JK184 in combination with CAR T cells or ICBs were evaluated in vitro and in vivo.

**Results:**

We selected three small molecule drugs from a compound library, among which JK184 directly induces tumor cell apoptosis by inhibiting the Hedgehog signaling pathway, modulates B7-H3 CAR T cells to an effector memory phenotype, and promotes B7-H3 CAR T cells cytokine secretion in vitro. In addition, our data suggested that JK184 exerts antitumor activities and strongly synergizes with B7-H3 CAR T cells or ICBs in vivo. Mechanistically, JK184 enhances B7-H3 CAR T cells infiltrating in xenograft mouse models. Moreover, JK184 combined with ICB markedly reshaped the tumor immune microenvironment by increasing effector T cells infiltration and inflammation cytokine secretion, inhibiting the recruitment of MDSCs and the transition of M2-type macrophages in an immunocompetent mouse model.

**Conclusion:**

These data show that JK184 may be a potential adjutant in combination with CAR T cells or ICB therapy.

**Supplementary Information:**

The online version contains supplementary material available at 10.1186/s12967-023-03875-4.

## Introduction

In recent years, solid cancers like Breast cancer (BC) and Colorectal cancer (CRC) treatments have dramatically changed from surgery, chemotherapy, and radiation therapy to a combination of targeted therapy, including small molecule drugs, ICBs and adoptive cell therapy (ACT)[1, 2]. Although much progress has been made in treatment modalities, patients with BC and CRC still suffer from significant treatment failures due to poor immune response. Consequently, it is urgent to explore novel treatment regimens.

Small molecule drugs with a small size (~ 500 Da) and suitable for oral administration have mostly been successfully applied to treat a wide range of cancers. The field of small molecule drugs covers kinases, epigenetic regulatory proteins, DNA damage repair enzymes, and proteasomes. To date, a total of 89 anticancer small molecules have been approved by the US FDA and the National Medical Products Administration (NMPA) of China [3, 4]. Among these small molecule drugs, some have induced complete cancer regression, while others have caused cancer cells to evolve and generate resistance immediately after cancer regression. Thus, developing revolutionary new drugs or repurposing "old" drugs urgently needs to be addressed.

ICB has been developed for the treatment of multiple tumor types. In particular, blocking the interaction between programmed cell death protein 1 (PD1) and its ligand (PD-L1) has substantially improved the prognosis of some patients [5, 6]. However, fewer than 40% of cases across multiple cancer types benefit from ICB treatments, and the underlying mechanism is not well understood [7–9]. Several studies have shown that IFN-γ from tumor-infiltrating lymphocytes (TILs) can upregulate the expression of PD-L1 and further inhibit the function of CD8 positive T cells [10–12]. PD-L1 regulation is critical for the improvement of anti-PD-L1/PD1 therapy. Moreover, recent studies suggested that combining small molecule inhibitors with ICB might increase treatment efficacy in patients, such as CDK4/6 inhibition augmenting antitumor immunity by enhancing T cell activation [13–15].

CAR T cell therapy as a main ACT approach has demonstrated great progress in hematological malignancy therapy [16–18]. Although CAR T cells have shown promise as a therapeutic for solid tumors, their effect on solid tumors lags behind that on hematological malignancies. How to mitigate tumor antigen heterogeneity, disrupt the structure of the tumor microenvironment (TME) and encourage T cell proliferation in solid tumors need to be addressed in CAR T cell therapy. Studies have demonstrated that multiple combination therapeutic strategies, including radiotherapy/chemotherapy [19, 20], immune checkpoint therapy [21, 22] and oncolytic viruses [23, 24], can effectively improve the efficacy of CAR-T therapy. Moreover, small molecule inhibitors like histone deacetylase inhibitors [25], IGF1R inhibitors [26], and DNA methyltransferase inhibitors [27, 28] combined with CAR-T cells have shown stronger antitumor effects.

In our recent study, we aimed to screen out novel small molecule drugs that can promote immunotherapy. We conducted a small molecule compound library screen in a human BC cell line to identify drugs whose addition contributes to B7-H3 CAR T cell killing. B7-H3, also known as CD276, is an immune checkpoint molecule that belongs to the B7 superfamily and has demonstrated wide overexpression in human malignancies but limited expression in normal tissues [29, 30]. We screened three small molecule drugs from a compound library named 3957 (BML284), 6014 (Picropodophyllin, PPP), 6114 (JK184), which had been previously reported as a Wnt signaling activator [31], an insulin-like growth factor 1 receptor (IGF1R) inhibitor [32], a Hedgehog inhibitor [33, 34] respectively. From in vitro and in vivo data, we found JK184 has both a tumor-suppressing and T cell-promoting role. We focused on assessing the effect of JK184 on several solid cancer type cells and activation of multiple intracellular signaling pathways in human B7-H3 CAR T cells, and antitumor activity of B7-H3 CAR T cells toward several types of solid cancers by performing in vitro coculture studies and in clinically relevant mouse models. Additionally, we tested whether the combination of JK184 and ICB treatment contribute to decrease tumor burden in immunocompetent mouse models.

## Materials and methods

### Animals

Six to eight-week-old female mice, including sixty C57BL/6, twenty-four BALB/c and forty immunodeficient M-NSG (NOD-Prkdc^scid^IL2rg^em1^/Smoc) mice, were purchased from the Model Animal Resource Information Platform of Nanjing University, China. Mice were housed in specific pathogen-free conditions, and all animal experiments followed a protocol approved by the Institutional Animal Care and Use Committee of Sichuan University.

### Cell lines and cell culture

The human cell lines HEK293T, MDA-MB-231, and HCT116 were obtained from American Type Culture Collection (ATCC). The murine cell line 4T1 was originally purchased from ATCC and MC38 was a kind of gift from Doctor Gu in our laboratory. MDA-MB-231-FFluc and HCT116-FFluc cell lines were produced by infection with the pLenti-CMV-luc2-IRES-Puro virus. HEK293T, HCT116, 4T1 and MC38 cells were cultured in DMEM medium (Gibco) supplemented with 10% fetal bovine serum (BI), 100 U/mL penicillin, and 100 mg/mL streptomycin (HyClone) at 37 ℃ with 5% CO_2_. MDA-MB-231 cells were maintained in RPMI-1640 medium (Gibco), supplemented as described above.

### T cell transduction

As described previously, a lentiviral vector expressing the second-generation anti-B7-H3 CAR was developed [30]. Lentivirus particles were produced by 293 T cells transiently transfected with the CAR T vector and the psPAX2 and pMD2G packaging plasmids. The lentiviral supernatants were collected, filtered with 0.45 mm filters, concentrated by ultracentrifugation at 25,000 g for 2 h, resuspended and immediately stored at − 80℃ until further use.

Human peripheral blood mononuclear cells (PBMCs) were isolated by density gradient centrifugation at a speed of 800 g for 15 min. T cells were activated with 100 U/ml IL-2 (PeproTech, Cat# 200–02) and T Cell TransAct™ (Miltenyi Biotec, Cat# 130–111-160). Then, continuous culture was performed using X-vivo medium (Lonza) supplemented with 10% FBS, 100 U/ml human IL-2 and 5 ng/ml IL15 (PeproTech, Cat# 200–15).

After 2 days, activated T cells were added to the plates coated with RetroNectin (TaKaRa, Cat# T100A/B), loaded with lentivirus particles by centrifugation at 1000 × g for 2 h at 4 °C, and centrifuged at 300 × g for 30 min. The following day, the transduced cells were collected, transferred to new plates and grown with fresh medium in the presence of IL-2 and IL15.

### Cytotoxicity assays

The activity of B7-H3 CAR T cells after coculture with tumor cells was assessed using ^51^Cr assays as previously described [30]. The ^51^Cr assay was adopted to detect the cytotoxicity of B7-H3-redirected CAR T cells. B7-H3 CAR T cells and tumor cells were labeled with sodium chromate (molecular formula: Na251CrO4) and cocultured at an E:T ratio of 4:1, 1:1, and 1:4 for 4 h. The supernatants were collected, and the radioactivity was measured using a gamma counter. The percentage of specific lysis was calculated by the following formula: (test release—spontaneous release)/(maximal release—spontaneous release) × 100.

### Screening and viability assays

For the screening assay, 10,000 MDA-MB-231-FFluc cells/well were seeded onto 96-well plates and cultured overnight. The following day, one strategy was the tumor cells directly incubated with compounds (TOPSCIENCE, L4000) at 1 µM for 48 h, another strategy was 40,000/well B7-H3 CAR T cells were added into plates with compounds at 1 µM for 48 h. The relative MDA-MB-231-FFluc cell activity in both strategies was quantified by measuring luminescence using a CLARIOstar plate reader (BMG LABTECH, Germany). Moreover, to detect the effect of the drug on the CAR T cells, the B7-H3 CAR T cells (10,000/well) were treated with compounds at 1 µM for 24 h individually, and the differentiation phenotype of T cells was analyzed by FACS.

### Cell proliferation assay

The 3-(4,5-dimethylthiazol-2-yl)-2,5-diphenyltetrazolium bromide (MTT) assay (Sigma–Aldrich, Cat# CT02) was used to measure the effect of the small molecule compound on cell viability according to the manufacturer's protocol. In brief, 3 × 10^3^ cells were seeded into 96-well culture plates, incubated overnight, dissolved compounds of interest in an appropriate solvent and treated with compound for 48 h. Next, 20 µl MTT solution (5 mg/mL) was added and incubated for 4 h. After the medium was replaced with 150 µl dimethyl sulfoxide (DMSO) (Sigma–Aldrich, Cat# D2650), the absorbance was measured using a microplate reader at 570 nm. T cells treated with different concentrations were stained with 0.2% trypan blue, and the number of trypan blue-positive and trypan blue- negative cells were counted by the Countstar Rigel S5. Each experiment was performed in triplicate.

### Coculture assays

Two-dimensional (2D) and three-dimensional (3D) tumor cells were cocultured with B7-H3 CAR T cells and drugs. In the two-dimensional (2D) cell coculture model, tumor cells were cocultured with CD19 CAR T cells or B7-H3 CAR T cells at an E:T ratio of 1:1, together with 1 μM drugs or not, which were simultaneously monitored by the xCELLigence® real-time cell analyzer (ACEA Biosciences). Three-dimensional (3D) spheroid models of HCT116 and HCT116-mCherry cells were developed to evaluate the tumor inhibition of DMSO, drugs (BML284/PPP/JK184), B7-H3 CAR T cells, drugs combined with B7-H3 CAR T cells. The 3D tumor spheroids were prepared by seeding HCT116 or HCT116-mCherry cells in ultralow attachment dishes (Corning) and grown in DMEM/F12 medium (Gibco) supplemented with 1% hormone mixture B27 (Thermo Fisher Scientific, Cat# 17504044), 10 ng /mL human recombinant epidermal growth factor EGF (Sino Biological, Cat# 10605-HNAE), and 10 ng /mL human recombinant fibroblast growth factor FGF (Sino Biological, Cat# 10573-HNAE) for 1 week. Subsequently, the tumor spheroids were transferred into a 24-well plate and incubated with DMSO, drugs, B7-H3 CAR T cells, and drugs plus B7-H3 CAR T cells respectively. B7-H3 CAR T cells were stained with CFSE (Biolegend, Cat# 423801). The HCT116 spheroids were stained with Calcein/PI (Beyotime, Cat# C2015M). Images were captured at 24 h after coculturing by a confocal microscope (Zeiss 880).

### Cell apoptosis

The Annexin V-FITC/7AAD Kit (Meilunbio, Cat# MA0428) was used for apoptotic analysis. MDA-MB-231 and HCT116 cells were incubated with media supplemented with DMSO or with drugs (BML284/PPP/JK184). After 24 h of incubation, the cells were collected and measured via flow cytometry.

### Flow cytometry

For drug-treated T cells, CD3, CD45RO and CD62L were used to determine the phenotype of T cells. T cells were incubated with antibodies in the dark at 4 °C or 30 min. For the coculture assay, T cells were fixed and stained with the Transcription Factor Buffer Set following the manufacturer's instructions, and Perforin and Granzyme B were then stained.

For mouse tumor tissues, MC38-xenograft tumors were digested into single-cell suspensions by rapid and gentle stripping, cut into small pieces and incubated with collagenase type IV (Sigma–Aldrich, Cat# C4-BIOC) and DNase-I (Roche, Cat# 10,104,159,001) at 37 °C for 1 h. Tumor digests were filtered through a 70 μm cell strainer (BD Falcon), and blocked with CD16/CD32 antibody, and dead cells were excluded by a Zombie NIR™ Fixable Viability Kit. Cells were stained with CD45, CD3 or CD4, CD8a, CD11b, F4/80, CD86, CD206, GR-1, FOXP3, IFN-γ and Granzyme B. All flow cytometry antibodies were from BioLegend and BD. The antibodies for flow cytometry are summarized in Additional file 1: Table S1. Data were collected with an LSR Fortessa cytometer (BD) and analyzed by FlowJo (v.10.6.1) software.

### RNA-sequencing analysis

Triplicate MDA-MB-231 cells were treated with 1 μM drugs (BML284/PPP/JK184) or DMSO for 24 h and then sorted and lysed in TRIzol reagent (Thermo Fisher Scientific, Cat# 15,596,018). Similarly, T cells from 3 donors were stimulated with T Cell TransAct™, and activated T cells were treated with drugs (1 μM) or DMSO for 24 h. Then cells were sorted and lysed in TRIzol reagent. Transcriptomic sequencing and analyses were conducted by Shanghai OE Biotech Co., Ltd. GO enrichment and KEGG pathway enrichment analyses of differentially expressed genes were performed using R based on the hypergeometric distribution.

### Gene set enrichment analysis (GSEA)

Raw data from the RNA sequencing analysis were normalized in three replicates for GSEA. We used the 4.1.0 version of the GSEA tool http://www.broadinstitute.org/gsea/index.jsp, and the REACTOME and KEGG sets were used for GSEA independently.

### Quantitative real-time PCR (qPCR)

qPCR was performed to validate the changes in gene expression identified by RNA-seq. Total RNA was extracted using the reagent TRIzol and reverse transcription reactions were performed using the PrimeScript RT reagent Kit (Takara, Cat# RR047A) according to the manufacturer's instructions. Target genes were amplified by SYBR Green Master Mix (Vazyme, Cat# Q411-02), and reactions were run on a real-time PCR system. The indicated gene expression was normalized to GAPDH. The sequences of the primers are listed in Additional file 1: Table S2.

### Immunofluorescence and immunohistochemistry

Cells were seeded and grown in 24-well culture plates overnight and incubated with DMSO or drugs (1 μM) for 24 h. Then, the cells were washed with PBS, fixed with 4% paraformaldehyde and treated with 1% Triton X-100 (Sigma–Aldrich, Cat# T8787) in PBS. Cells were stained with Actin-Tracker Green (Beyotime, Cat# C2201S) to visualize F-actin, and nuclei were visualized by staining with DAPI (Solarbio, Cat# C0060). Images were captured by a confocal microscope (Olympus IXplore SpinSR).

For Immunohistochemistry (IHC), tumors were embedded in formalin, fixed in paraffin, and then sectioned at a thickness of 4 μm. Sections were deparaffinized. Antigen was retrieved in 10 mM citrate buffer and blocked with PBS containing 10% normal goat serum, followed by incubation with primary antibody at 4℃ overnight. After staining, sections were washed and incubated with goat anti-rabbit secondary antibodies, followed by diaminobenzidine (DAB) and counterstaining with hematoxylin (ZSGB-Bio). Images were detected and captured using a fluorescence microscope (DP80, Olympus).

### In vivo treatments

In the immunodeficient mouse model, 2 × 10^6^ MDA-MB-231-FFluc or HCT116-FFluc cells were subcutaneously injected into mice. 1 × 10^7^ B7-H3 CAR T cells were intravenously administered to mice on days 7 and 14, and 5 mg/kg JK184 or an equal volume of PBS control was injected intraperitoneally for 10 days beginning on day 7. Drug dosing was selected based on published literature [33]. The progression of tumors was confirmed by bioluminescence signals and mouse body weight was measured by a Vernier caliper. The IVIS imaging system (Caliper Life Sciences) operation method and the bioluminescence signal were carried out as previously described [30].

In the immunocompetent mouse model, each mouse was subcutaneously injected with 1 × 10^5^ MC38 or 4T1 cells in the right flank. JK184 (5 mg/kg) was intraperitoneally administered to mice on days 7 to 17. 100 μg of murine anti-PD1 antibody or murine anti-CTLA4 antibody or its isotype-matched control antibody was intraperitoneally administered to mice on days 7 and 10. The murine anti-PD1 antibody (BioXcell, Cat# BE0146), murine anti-CTLA4 antibody (BioXcell, Cat# BE0131) and its isotype-matched control antibody (BioXcell, Cat# BE0089) were purchased from Bio X Cell. Body weight was measured every 3 days. Tumor size was confirmed by Vernier caliper measurement (tumor size = long diameter × (short diameter)^2^/2).

### Statistics

Statistical analysis was performed using GraphPad Prism 9. Unpaired two-tailed Student’s t test was used to compare two groups of independent samples. Survival curves were analyzed by using the log-rank test. Levels of significance were set at *p < 0.05, **p < 0.01, ***p < 0.001 and ****p < 0.0001. Error bars represent the mean ± standard deviation (SD).

## Results

### Generation of B7-H3 CAR-T cells and functional tests in vitro

In our previous studies, B7-H3 was found to be wildly expressed in human tumor cells, and B7-H3 CAR T cells have been developed against multiple tumors [5, 30]. Although significant antitumor efficacy was observed in vitro, there remains a certain distance to achieve a cure in B7-H3 CAR T cell therapy against solid tumors. In this study, we aimed to screen drugs to enhance immunotherapy efficacy. First, second-generation B7-H3 CAR T cells were developed, which comprised a single-chain variable fragment (scFv) derived from our library [30] and included 4-1BB and CD3ζ endodomains (Additional file 1: Fig. S1A). mCherry was inserted as a tracker for detecting the expression of CAR, which was measured using fluorescence microscopy (Additional file 1: Fig. S1B) and Fluorescence-activated cell sorting (FACS) (Additional file 1: Fig. S1C). Second, the expression level of B7-H3 in a BC cell line (MDA-MB-231) and CRC cell line (HCT116) was examined by FACS using B7-H3-PE (BioLegend, Cat# 331,606) as the primary antibody (Additional file 1: Fig. S1D). Finally, we assessed the cytolytic activity of B7-H3 CAR T cells, and CD19 CAR T cells were used as a negative control in the coculture system. We found that B7-H3 CAR T cells could efficiently lyse cancer cells at an effector:target (E:T) ratio of 4:1 at 24 h but under 20% lysis at an E:T ratio of 1:4 (Additional file 1: Fig. S1E).

### The schema of the screening assay

**Fig. 1:**
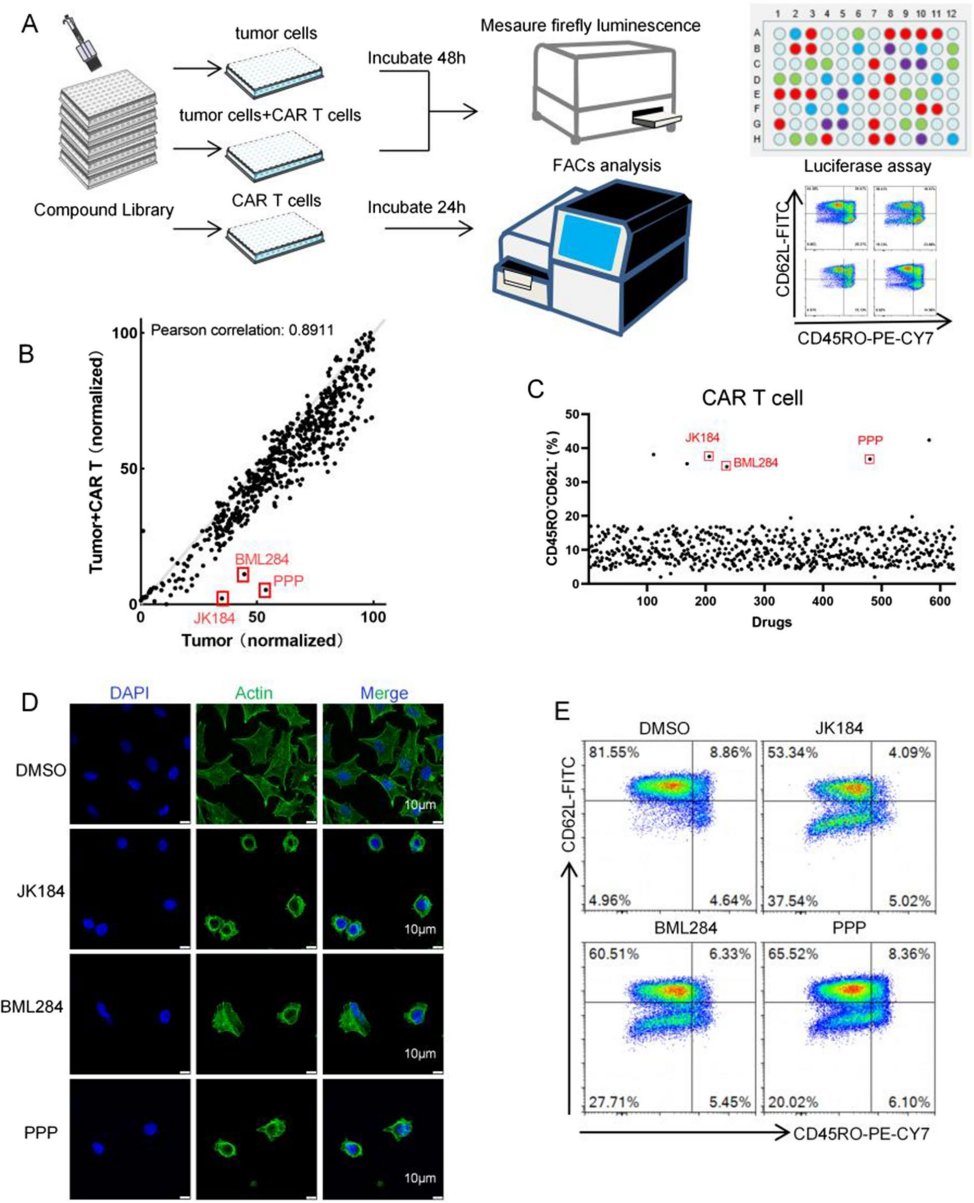
A small molecule drug screening for enhancement of CAR T cell antitumor function. **A** Schematic representation of the screening process, which was divided into three parts. MDA-MB-231-FFluc cells (10,000/well) were treated with small molecules (1 µM) for 48 h. MDA-MB-231-FFluc cells (10,000/well) were simultaneously cocultured with B7-H3 CAR T cells (40,000/well) and small molecules (1 µM) for 48 h. B7-H3 CAR T cells (10,000/well) were treated with small molecules (1 µM) for 24 h. The first two groups of MDA-MB-231-FFluc cell activity were detected by measuring luminescence. The phenotype assessment of B7-H3 CAR T cells was analyzed by FACS in the last group. **B** Scatter plot showing the level of firefly luminescence in the first two groups. The values on the X and Y axes are the normalized signal values of the group (log2 scaled). **C** Scatter plot showing the CD45RO^−^CD62L^−^ phenotype of CAR T cells after coincubation with 624 small molecule drugs for 24 h. The red dots represent BML284/PPP/JK184, which showed promising antitumor effects and increased B7-H3 CAR-T cells activity. **D** The morphology of BML284/PPP/JK184-treated MDA-MB-231 cells, which were exhibited by labeling with F-actin. Scale bars, 10 µm. **E** The differentiation phenotype of B7-H3 CAR T cells after coincubation with BML284/PPP/JK184 for 24 h. Each experiment was performed in triplicate

To identify compounds that promote CAR T cell antitumor activity, a small molecule library of 624 unique compounds (TOPSCIENCE, L4000) was screened. The screening process is summarized in a flow diagram (Fig. 1A). Briefly, MDA-MB-231-FFluc cells, B7-H3 CAR T cells, and MDA-MB-231-FFluc cells cocultured with B7-H3 CAR T cells at an E:T ratio of 1:4, were treated with small molecules (1 μM) for 24–48 h. The relative MDA-MB-231-FFluc cell activity was quantified by measuring luminescence. The drug-treated groups of MDA-MB-231-FFluc cells and MDA-MB-231-FFluc cocultured with B7-H3 CAR T cells were detected for firefly luminescence by a CLARIOstar plate reader (BMG LABTECH, Germany). In the drug-treated B7-H3 CAR T cell group, T cell markers (CD45RO and CD62L) were analyzed by FACS. The screening data are shown in Fig. 1 B and C. Among the three screening groups, three of our most potent hits were 3957(BML284), 6014(Picropodophyllin, PPP), 6114(JK184). After treatment with BML284/PPP/JK184 (1 μM) for 24 h, MDA-MB-231 cells exhibited shrunken morphology and the images were captured on a confocal microscope (Fig. 1D). B7-H3 CAR T cells showed an effector memory phenotype after treatment with BML284/PPP/JK184 (1 μM) for 24 h (Fig. 1E).

### Identification of BML284/PPP/JK184 as potent adjutants of CAR T cells

**Fig. 2:**
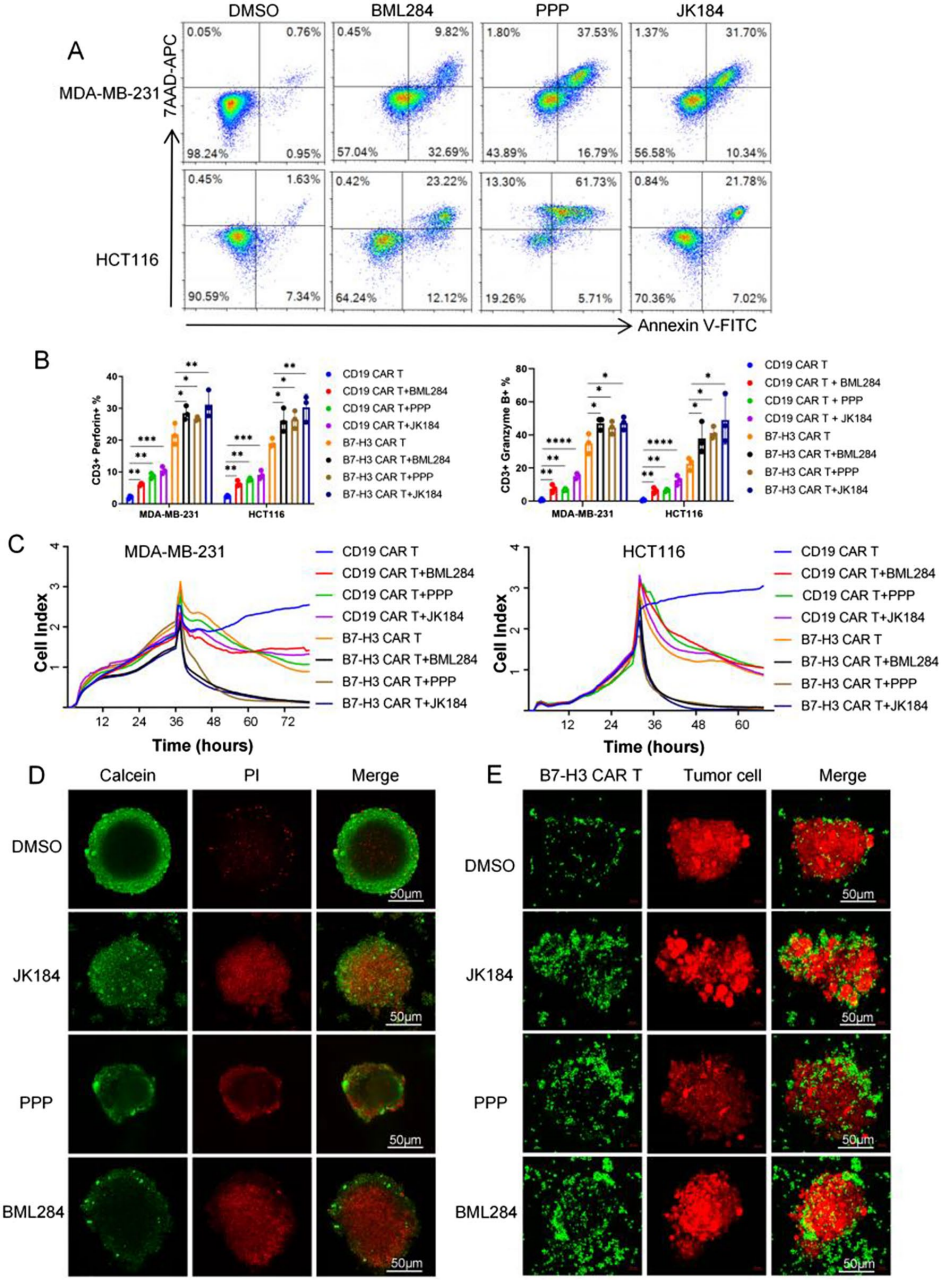
Identification of BML284/PPP/JK184 as a potent adjutant of CAR T cells in vitro. **A** MDA-MB-231 and HCT116 cells were analyzed by flow cytometry for Annexin V/7AAD staining after treatment with BML284/PPP/JK184 (1 µM) for 24 h. **B** CD19 or B7-H3 CAR T cells cocultured with tumor cells at an E:T ratio of 1:1 combined with BML284/PPP/JK184 or DMSO. The secretion of perforin and granzyme B by CAR T cells was detected and compared. **C** The cytotoxicity of multiple treatment groups on MDA-MB-231 and HCT116 cells in this coculture assay was monitored using the RTCA system. **D** and **E** HCT116 spheroids were incubated with DMSO, BML284/PPP/JK184, B7-H3 CAR T cells, and BML284/PPP/JK184 plus B7-H3 CAR T cells respectively, and pictures were captured by a confocal microscope (Zeiss 880). Scale bars, 50 µm. In Fig. 2C, *P < 0.05, ***P < 0.001, by a two-tailed Student’s t test. Each experiment was performed in triplicate

To assess the functional relevance of the tumor cells and T cells induced by BML284/PPP/JK184 respectively, the antitumor activity of BML284/PPP/JK184-treated B7-H3 CAR T cells against cancer cells or BML284/PPP/JK184-treated cancer cells was tested in vitro. Therefore, we first evaluated the in vitro antitumor effects of BML284/PPP/JK184 on MDA-MB-231 and HCT116 cells at different concentrations. The half-maximal inhibitory concentrations (IC50) of JK184 were 78.68 nM (MDA-MB-231) and 95.56 nM (HCT116), BML284 were 293.3 nM (MDA-MB-231) and 237.9 nM (HCT116), 6014 were 29.3 nM (MDA-MB-231) and 111.9 nM (HCT116) (Additional file 1: Fig. S2A). To make the drug effects more apparent and facilitate comparison, we have tested the drugs at a concentration of 1 μM. Then, the apoptosis of MDA-MB-231 and HCT116 cells was analyzed by FACS. As shown in Fig. 2A, after treatment with 1 µM drugs (BML284/PPP/JK184) for 24 h, the percentage of early apoptosis plus late apoptosis cells was approximately 42.51%/54.32%/42.04% in MDA-MB-231 cells and 35.34%/66.83%/28.8% in HCT116 cells. The results indicated that BML284/PPP/JK184 could induce MDA-MB-231 and HCT116 cell apoptosis. We noticed that the expression of B7-H3 was not influenced with BML284/PPP/JK184 treatment (Additional file 1: Fig. S2B).

Next, to gain better insight into the effect of BML284/PPP/JK184 on T cells, we measured the T cell survival rate after treatment with BML284/PPP/JK184 at different concentrations. We observed that lower concentration of BML284/PPP/JK184 (1 µM) showed non-significant influence on T cell survival (Additional file 1: Fig. S2C). In addition to the analysis of phenotypic changes in activated T cells treatment with BML284/PPP/JK184 (1 µM), we also examined the expression levels of the canonical T cell activation and exhaustion markers such as CD69, CD25, PD-1, and LAG-3 using FACS. Flow cytometric analysis showed that BML284/PPP/JK184 treatment did not cause any significant change in the expression of the T cell markers CD25 and CD69 (Additional file 1: Fig. S2D). Interestingly, the expression of PD1 and LAG3 was declined after treatment with JK184 in B7-H3 CAR T cells and T cells (Additional file 1: Fig. S2E and S2F). Moreover, we also tested the phenotypic changes in naive T cells treated with BML284/PPP/JK184 (1 µM), and the results were similar to that of activated T cells (data not shown).

In the coculture assay, to reflect the synergistic killing effect of BML284/PPP/JK184, we set a low effector to target (E:T) ratio of 1:1 and observed a significant killing effect of B7-H3 CAR T cells against MDA-MB-231 and HCT116 cells when combined with BML284/PPP/JK184 (1 µM) after coincubation for 24 h. In addition, the secretion of perforin and granzyme B by T cells was also analyzed with FACS (Fig. 2B). B7-H3 CAR T cells with or without drugs mediated antitumor activity, yet the secretion level of perforin or granzyme B increased dramatically only in the BML284/PPP/JK184 combination group. The residual tumor cells were estimated with crystal violet staining, and the results are presented in Additional file 1: Fig. S2G. Moreover, we monitored the cytotoxicity of multiple treatment groups on MDA-MB-231 and HCT116 cells using the Real-Time Cellular Analysis (RTCA) system (Fig. 2C). Similarly, the antitumor effects of the BML284/PPP/JK184+B7-H3 CAR T cell combination treatment group were dramatically enhanced compared to those of the B7-H3 CAR T cell (p < 0.0001, p < 0.0001, p < 0.0001, respectively), BML284/PPP/JK184+CD19 CAR T cell (p < 0.0001, p < 0.0001, p < 0.0001, respectively) and CD19 CAR T cell (p < 0.0001, p < 0.0001, p < 0.0001, respectively) groups. To partially mimic the compactness of solid tumors, we established a three-dimensional (3D) spheroid model of HCT116 cells. Calcein/PI staining showed that BML284/PPP/JK184 induced HCT116 spheroid death (Fig. 2D). In Fig. 2E, B7-H3CAR T cells were primarily stained with CFSE, and HCT116-mCherry cells were employed. It was directly observed that more B7-H3 CAR T cells were deposited in the peripheral rim of tumor spheroid regions and jointly attacked the tumor spheroid in the combination treatment group. In summary, 3957/6014/JK184 combined with B7-H3 CAR T cells has a better antitumor effect than BML284/PPP/JK184 or B7-H3 CAR T cells alone.

### The mechanism by which BML284/PPP/JK184 acts on MDA-MB-231 cells

**Fig. 3:**
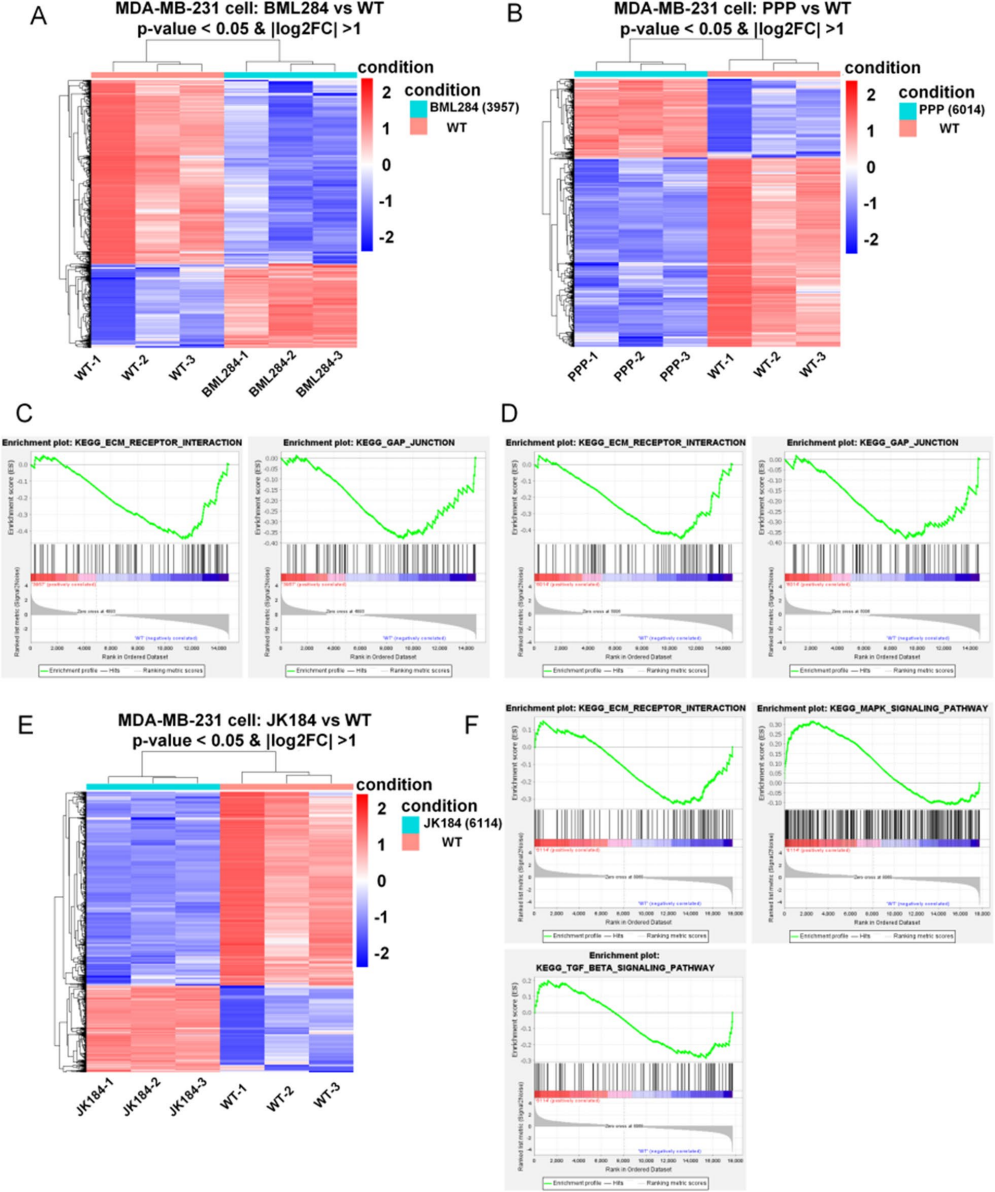
Transcriptional signatures of MDA-MB-231 after BML284/PPP/JK184 treatment. **A**, **B** and **E** Hierarchical clustering of the RNA-seq analysis results shows differentially expressed genes between BML284/PPP/JK184-treated MDA-MB-231 cells and nontreated MDA-MB-231 cells (n = 3). **C** and **D** Representative GSEA enrichment plot demonstrating significant downregulation of ECM-receptor interaction pathway-related genes (BML284, FDR = 0.705 and NES = − 1.452, PPP, FDR = 1.0 and NES = − 1.463) and gap junction pathway-related genes (BML284, FDR = 0.12 and NES = − 1.248, PPP, FDR = 1.0 and NES = − 1.225) in BML284/PPP-treated MDA-MB-231 cells versus nontreated MDA-MB-231 cells (WT). **F** GSEA analysis highlighted the ECM-receptor interaction pathway (FDR = 0.749 and NES = − 1.374), the MAPK pathway (FDR = 0.127 and NES = 1.569), and the TGFβ pathway (FDR = 0.928 and NES = − 1.177) alternations in JK184-treated MDA-MB-231 cells versus nontreated MDA-MB-231 cells (WT)

To further explore the molecular mechanism involved in the effect of BML284/PPP/JK184 in MDA-MB-231 cells, RNA-sequencing assays were performed. As expected, the RNA sequencing of MDA-MB-231 cells treated with BML284/PPP/JK184 identified 494/419/493 highly upregulated and 1081/1008/1097 significantly downregulated genes. In Fig. 3 A, B and E, the RNA sequencing data were shown by heat maps, which displayed different gene expression profiles among samples after treatment with control or BML284/PPP/JK184. Meanwhile, we applied gene set enrichment analysis (GSEA) to identify the changed phenotype of MDA-MB-231 cells. GSEA demonstrated that both extracellular matrix-receptor (ECM-receptor) interaction pathway and gap junction pathway were significantly enriched in cells treated by BML284 or PPP (Fig. 3C, D). In agreement with above results, in JK184-treated cells, we found reduced expression of ECM-receptor interaction pathway genes and a lower ECM-receptor interaction output score compared to control cells (Fig. 3F). Astonishingly, based on the GSEA enrichment analysis, the MAPK and TGFβ pathways which play critical roles in cell development were found to be significantly enriched in MDA-MB 231 cells treated by JK184 (Fig. 3F). JK184 activated more signaling pathways after acting on cells than the first two drugs did (p = 0.7048, p = 0.3179, respectively). Therefore, how JK184 regulated the concomitant activation of these pathways which drive tumor cells to be sensitively recognized and killed by T cells is interesting.

As expected, the RNA sequencing of MDA-MB-231 cells treated with JK184 identified the enriched pathways, such as breast cancer, Hedgehog signaling, and apoptosis, which were indicated by using the Kyoto Encyclopedia of Genes and Genomes (KEGG) analysis (Additional file 1: Fig. S3A). The enrichment results were consistent with previous studies, and we found that some highly expressed genes of the Hedgehog signaling pathway were inhibited after JK184 treatment, such as SMO, PTCH1, GLI1, GLI2, GLI3, and SUFU (Additional file 1: Fig. S3B). The expression of these genes was also confirmed by real-time PCR (Additional file 1: Fig. S3C).

### The mechanism by which JK184 acts on T cells

**Fig. 4:**
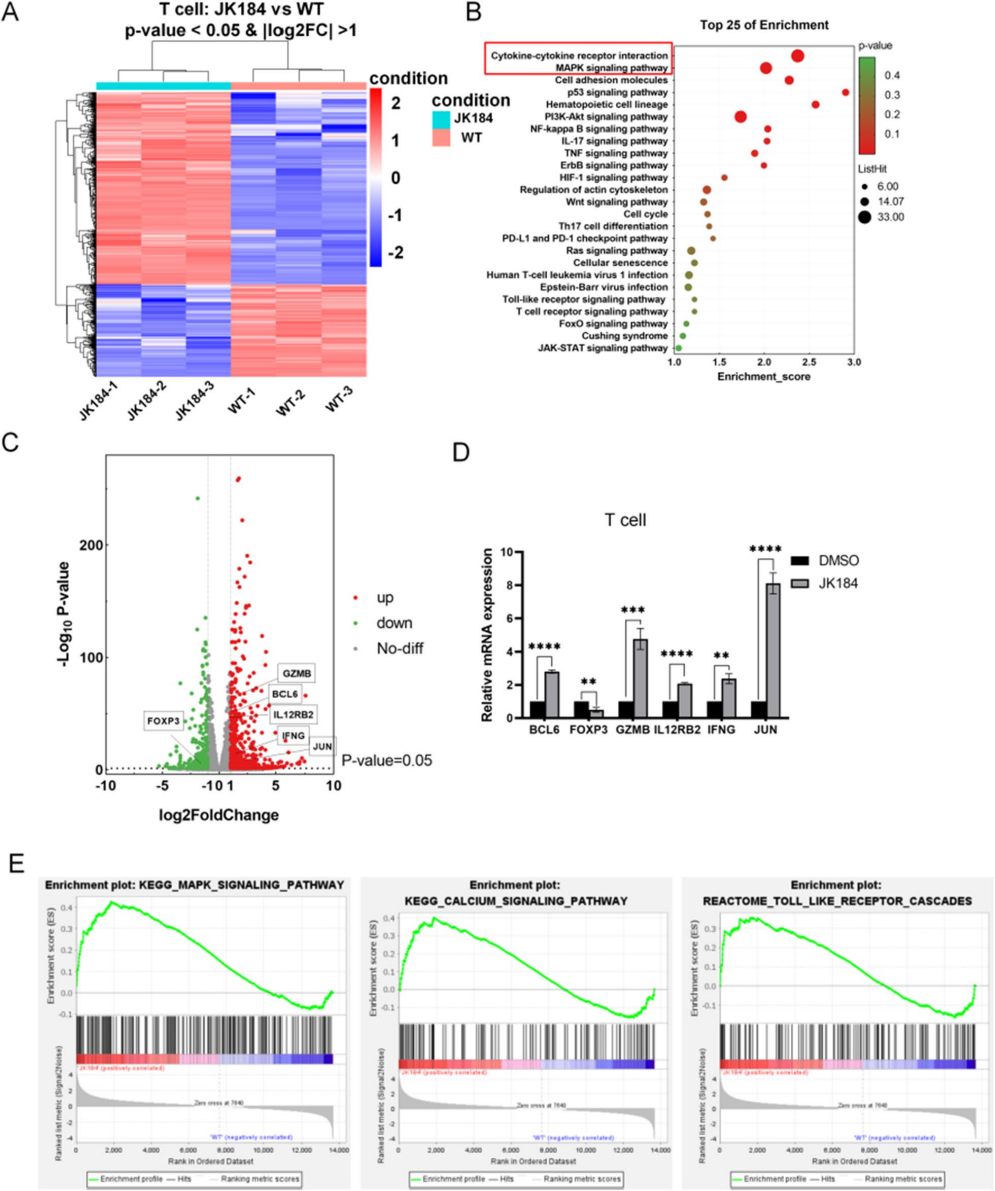
Transcriptional signatures of T cells after JK184 treatment. **A** Heatmap showing differentially expressed genes between JK184-treated T cells and nontreated T cells (WT). Each sample group comprised triplicates. **B** Bubble plot of the top 25 enriched KEGG pathways in JK184-treated T cells versus nontreated T cells. The size and color of the circles in the plots are scaled according to the enrichment score calculated by the number of differentially expressed genes and P value. **C** The volcano plot demonstrating fold changes in gene expression in JK184-treated T cells compared with nontreated T cells. Red dots and green dots represent significantly upregulated genes and downregulated genes respectively, and gray dots represent genes that are statistically nonsignificant. Values are presented as the log2 of the tag counts. **D** Real-time PCR to confirm the expression of genes marked in the volcano plot. Each sample group comprised triplicates. **E** Representative GSEA enrichment plot demonstrating significant upregulation of KEGG MAPK signaling pathway-related genes (FDR = 0.066 and NES = 1.615), KEGG calcium signaling pathway (FDR = 0.11 and NES = 1.471) and REACTOME Toll-like receptor cascade-related genes (FDR = 0.084 and NES = 1.733) in JK184-treated T cells versus nontreated T cells. T tests were used to determine statistical significance of the differences in (D). *P < 0.05, **P < 0.01, ***P < 0.001 and ****P < 0.0001

To the best of our knowledge, Hedgehog signaling pathway inhibitors have been developed for the treatment of cancer, but few studies have investigated the effects of Hedgehog inhibitors on immune cells or their potential to be combined with ICB-based immunotherapy. Interestingly, a recent study highlighted the relevance of Hedgehog signaling in immune cells, which described a novel immunometabolic function of Hedgehog signaling on macrophages [35]. Here, to detect transcriptomic changes in T cells under JK184 (1 µM) treatment, we performed mRNA profiling. Next, the RNA sequencing data were shown using a heat map (Fig. 4A), and a total of 761 genes were significantly changed, including 510 upregulated genes and 251 downregulated genes. From the KEGG analysis, the top 25 enrichment pathways are shown in Fig. 4B. Strangely, cytokine-cytokine receptor interactions and the MAPK signaling pathway were enriched, but the Hedgehog signaling pathway was not found. Meanwhile, we applied gene set enrichment analysis (GSEA) to identify the changed phenotype of T cells. A similar description of the gene sets, including upregulated MAPK signaling pathway-related genes, toll-like receptor cascade-related genes, and calcium signaling pathway genes, is provided in Fig. 4E. Interestingly, the analysis of differentially expressed genes showed that JUN, GZMB, IFNG, IL12RB2 and BCL6 were upregulated, whereas genes such as FOXP3 were downregulated (Fig. 4C), and the result was confirmed by real-time PCR in T cells treated with JK184 (Fig. 4D). Thus, JK184 induces tumor cell death through the inhibition of the Hedgehog signaling pathway and the concomitant activation of pathways. However, JK184 may increase the activity of T cells and influence critical components of the cytotoxic machinery in T cells by regulating the MAPK signaling pathway.

### Enhancement by JK184 of the antitumor activity of B7-H3 CAR T cells in vivo

**Fig. 5:**
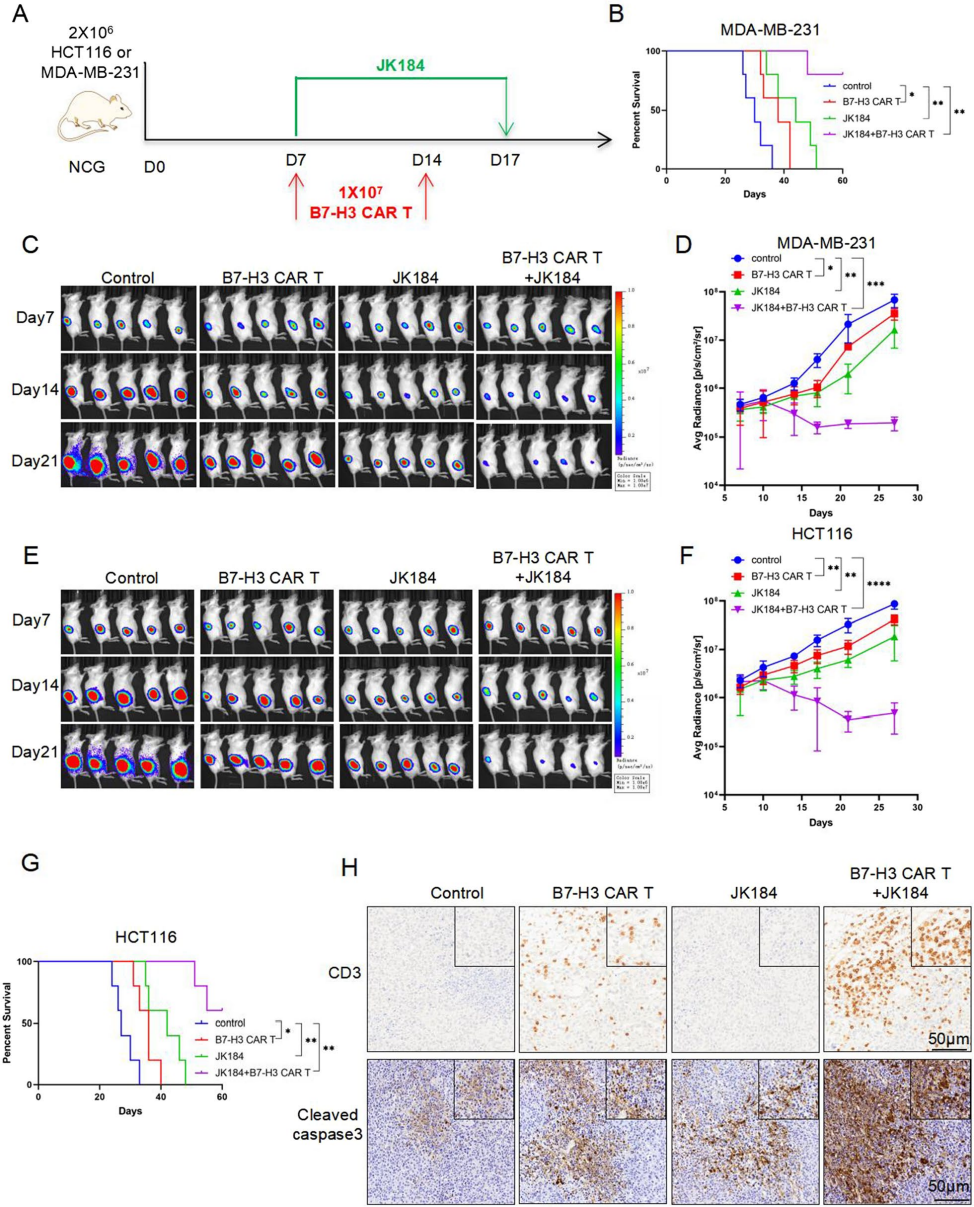
Combination treatment with JK184 and B7-H3 CAR T cells showed superior tumor control in MDA-MB-231 and HCT116 xenograft mouse models. **A** Treatment scheme used in the MDA-MB-231-FFluc and HCT116-FFluc xenograft models. **B** Kaplan-Meier analysis of MDA-MB-231-FFluc tumor-bearing mice in each group (n = 5 per group). **C** and **E** On days 7, 14, and 21 after tumor injection, representative bioluminescence images of MDA-MB-231-FFluc or HCT116-FFluc cell growth are shown. **D** and **F** Tumor growth was monitored by bioluminescence, and the average tumor radiance (p/s/cm^2^/sr) was calculated in the two xenograft models. **G** Overall survival analysis of HCT116-FFluc tumor-bearing mice in each group (n = 5 per group). **H** Mice-bearing tumors derived from MDA-MB-231-FFluc cells were sacrificed on day 20 after inoculation. Paraffin tumor sections were stained with CD3 and cleaved caspase3. Scale bar: 50 μm. T tests were used to determine statistical significance of the differences in **D** and **F**. *P < 0.05, **P < 0.01, ***P < 0.001 and ****P < 0.0001, ns not significant. P values for **B** and **G** were determined by the log-rank test, two-tailed, *P < 0.05, **P < 0.01

According to the preceding results, JK184 could improve the activity of T cells and promote antitumor effect of B7-H3 CAR T cells in vitro. Next, we tested the efficacy of the combinatorial therapy in vivo using xenograft NSG mouse models. Here, we adopted two xenograft tumor models. First, 2 × 10^6^ MDA-MB-231-FFluc or HCT116-FFluc cells were subcutaneously injected into the right flank of mice. 7 days after tumor inoculation, mice were injected intravenously with 1 × 10^7^ B7-H3 CAR T cells on days 7 and 14 respectively, and 10 consecutive days of JK184 treatment (Fig. 5A). Tumor burden was monitored by in vivo bioluminescence imaging (BLI) every 3 days, and tumor average radiance (p/s/cm^2^/sr) was calculated using Living Image. As shown in Figs. 5C, 4F, although B7-H3 CAR T cells treatment or JK184 treatment alone mediated significant regression of xenografts compared with the control, it was more effective, even completely regressing, in the combinatorial therapy. The overall survival of MDA-MB-231-FFluc or HCT116-FFluc tumor-bearing mice was significantly prolonged in the treatment groups, including B7-H3 CAR-T cells treatment, JK184 treatment and combinatorial therapy (Fig. 5B and G). Animal weights did not significantly change during the course of treatment (Additional file 1: Fig. S4A and S4B). Mouse-bearing tumors from MDA-MB-231-FFluc cells were sacrificed on day 20 after inoculation. To evaluate the presence and frequency of B7-H3 CAR T cells infiltrating the tumor, IHC of CD3 staining was performed. The results demonstrated that more CAR T cells had infiltrated into tumors in the combination group. Meanwhile, IHC of cleaved caspase3 staining predicated that the tumor cells underwent apoptosis in each group, and more apoptotic tumor cells were observed in the combination therapy group (Fig. 5H).

### The antitumor activity of JK184 in combination with ICB

**Fig. 6:**
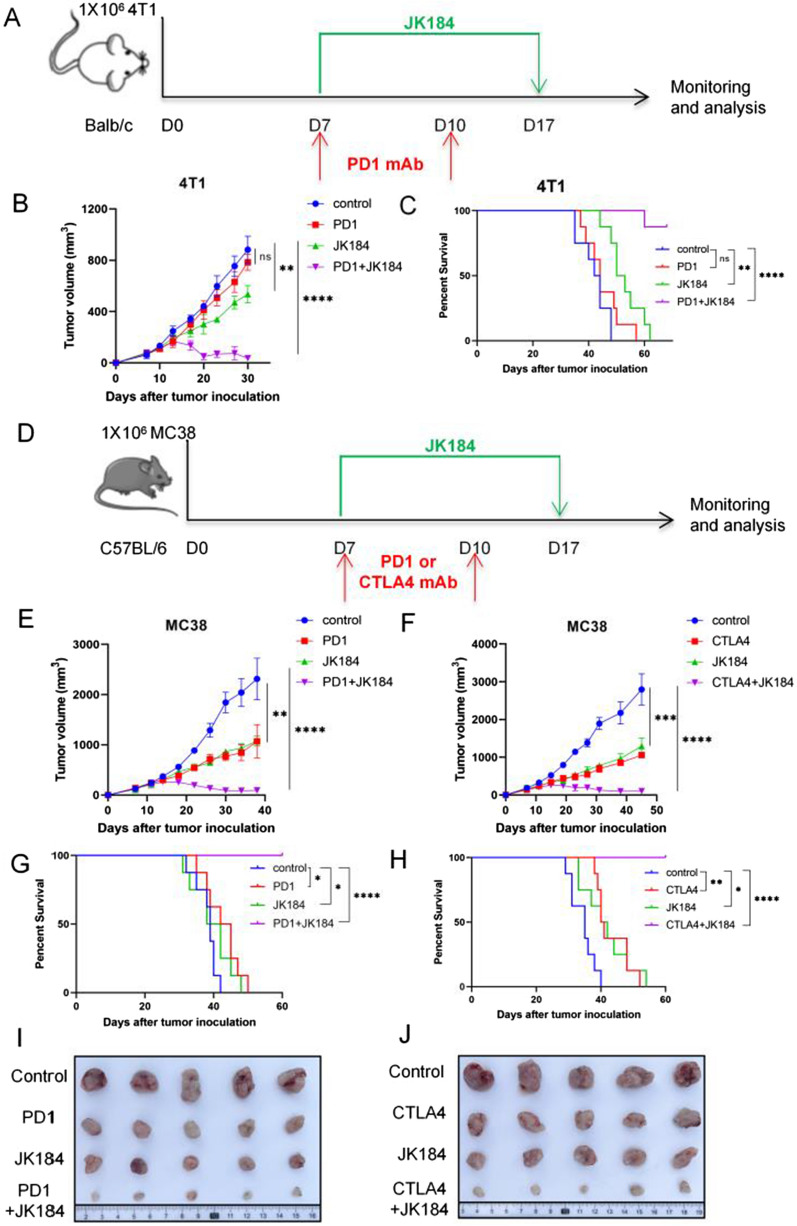
JK184 combined with ICB synergistically induces tumor regression. BALB/c mice were subcutaneously inoculated with 4T1 tumor cells, and C57BL/6 mice were subcutaneously inoculated with MC38 tumor cells. The above mice were sacrificed, and tumors were removed and photographed at the treatment endpoint. **A** Timeline of mouse models of 4T1 tumor cells with treatment schedules. **B** 4T1 tumor growth in the control group, PD1 group, JK184 group and PD1 plus JK184 treatment group (n = 6 mice per group). **C** Kaplan–Meier curves showing the overall survival of 4T1 tumor-bearing mice treated with either control, PD1, JK184, or PD1+JK184. **D** Diagram depicting the treatment schedule for the MC38 tumor model. In our models, JK184 was combined with PD1 or CTLA antibody for treatment. **E** and **F** MC38 tumor growth resulting from tumors treated with control, PD1 (CTLA4), JK184, or PD1 (CTLA4)+JK184. **G** and **H** Percent survival was analyzed using Kaplan–Meier analysis in the two models. **I** and **J** Representative tumor pictures are shown. T tests were used to determine statistical significance of the differences in **B**, **E** and **F**. **P < 0.01, ***P < 0.001 and ****P < 0.0001, ns not significant. P values for **C**, **G** and **H** were determined by the log-rank test, two-tailed, *P < 0.05, **P < 0.01, ****P < 0.0001, ns not significant

Generally, cell line xenograft models were used to target human tumor studies, but these immunodeficient models could not mimic the interplay of the endogenous immune system, and such xenograft models failed to recapitulate endogenous immune activation and detect severe toxicity in normal tissues. Here, we tested the antitumor activity of JK184 using 4T1 and MC38 mouse models. Mouse anti-PD1 or anti-CTLA4 monoclonal antibodies were used as combination reagents. The schemes of the 4T1 and MC38 mouse treatments are shown in Fig. 6A and D. Briefly, 1 × 10^5^ 4T1 or MC38 cells were subcutaneously injected into Balb/c or C57BL/6 mice. Then, tumor-bearing mice were divided into four groups: control (isotype-matched control antibody), ICB treatment, JK184 treatment, and ICB + JK184 treatment. Starting on day 7 and continuing through day 17, mice received JK184 treatment. On days 7 and 10, 100 µg anti-PD1 or anti-CTLA4 antibody was intraperitoneally injected into each mouse. Tumor volume was measured using a Vernier caliper every 3 days beginning on day 7. As shown in Fig. 6B, E and F, JK184 and JK184 combined with ICB mediated significant regression of 4T1 or MC38 tumors compared with the control, and the antitumor activity of combination therapy was superior to that of ICB or JK184 alone. In the 4T1 mouse model, although tumor cells displayed few responses to the anti-PD1 antibody treatment, it had made great regression of tumor burden in the anti-PD1 combination with JK184 therapy (p < 0.001) (Fig. 6B). Furthermore, the combination therapy significantly retarded the growth of the tumor and prolonged the survival of mice (Fig. 6C). In the MC38 mouse model, JK184, anti-PD1 or anti-CTLA4 antibody treatment inhibited tumor growth, but the combination treatment almost achieved complete tumor regression in some mice (Fig. 6E and F), and no mice died during the 60-day survival observation (Fig. 6G and H). The tumor volume calculation results and the corresponding solid tumor graph are shown in Fig. 6I, J, and the animal weights in the three models were measured (Additional file 1: Fig. S4C, S4D and S4E).

### JK184 combined with ICB promoted T cell infiltration and reshaped the TME

**Fig. 7:**
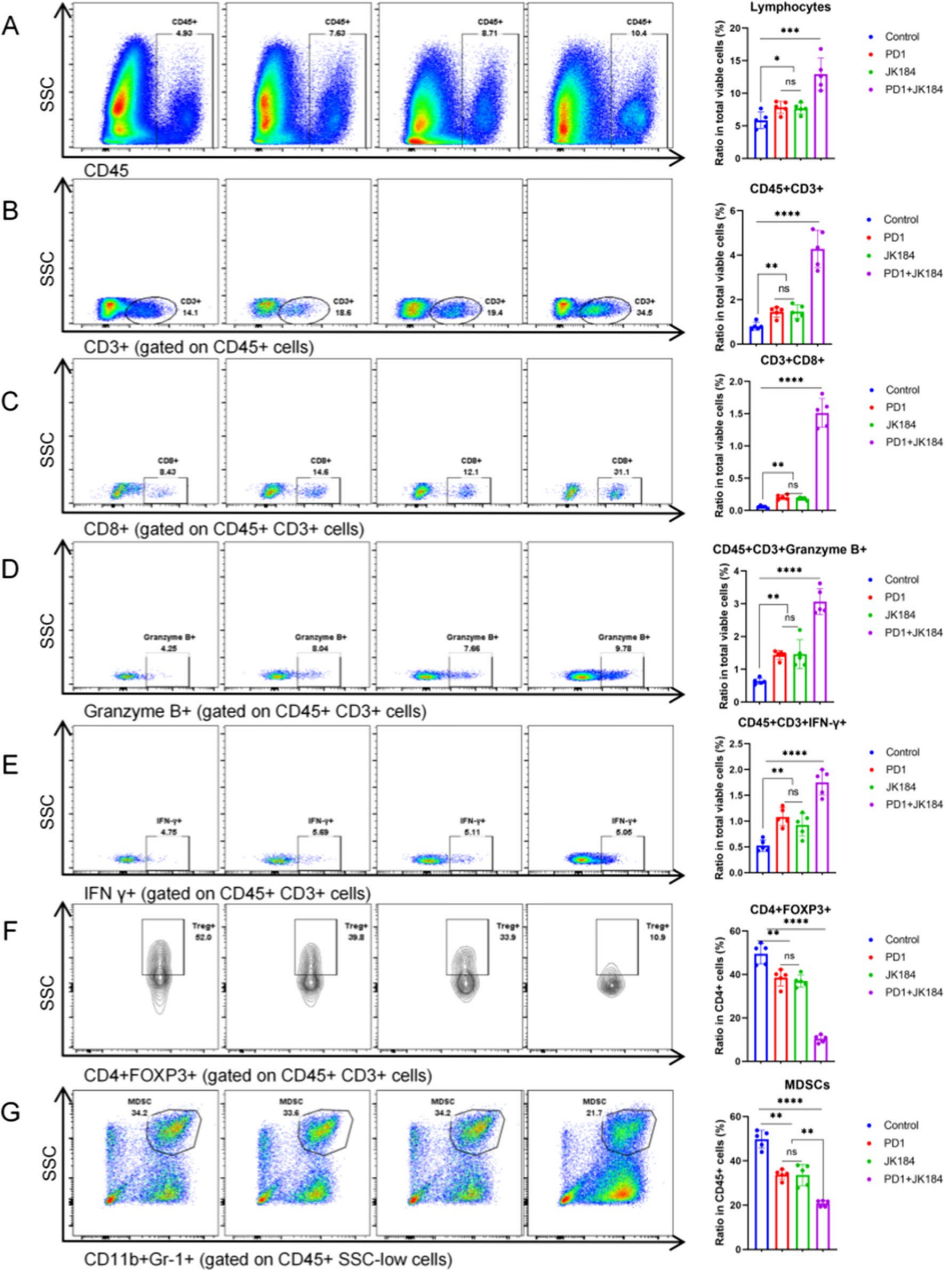
Flow cytometry assays to analyze the tumor immune microenvironment in PD1 combined with JK184 treated MC38 tumors. Representative images of **A** tumor-infiltrating lymphocytes, **B** CD3^+^ T cells, **C** CD8^+^ T cells, **D** granzyme B^+^ T cells, **E** IFN-γ^+^ T cells, **F** CD4^+^ FOXP3^+^ T cells, and **G** myeloid-derived suppressor cells (MDSCs). The relative quantitative analysis was performed by the ratio of tumor-infiltrating immune cells to total alive cells in the prepared cell suspension. T tests were used to determine statistical significance of the differences as indicated: *P < 0.05, **P < 0.01, ***P < 0.001 and ****P < 0.0001, ns not significant

To explore the effect of JK184 combined with PD1 in MC38 tumor-bearing mice, flow cytometry assays were used to analyze the tumor immune microenvironment. First, the number of tumor-infiltrating lymphocytes (TILs) was collected, and both PD1 and JK184 increased the density of TILs compared to control (p < 0.05). The combination treatment group had a significantly increased density of TILs compared to the control (p < 0.001) (Fig. 7A). In addition, the ratio of T cells in all viable cells was analyzed compared to the control, JK184 (p < 0.01), PD1 (p < 0.01), and JK184+PD1 (p < 0.0001) groups (Fig. 7B). Apart from the number of T cells, we further examined the cytotoxic activity of T cells and observed that JK184+PD1 simultaneously enhanced it. The number of CD8+T cells was elevated after JK184 (p < 0.01), PD1 (p < 0.01) or JK184+PD1 (p < 0.0001) treatment (Fig. 7C). Moreover, the densities of granzyme B+and IFN-γ+T cells were enormously elevated after JK184 + PD1 treatment relative to the control (p < 0.0001, p < 0.0001, respectively), and both were increased, which was statistically significant in the JK184 or PD1 treatment group (Fig. 7D and E).

On the other hand, we focused on the analysis of regulatory T (Treg) cells and myeloid-derived suppressor cells (MDSCs), which are two leading components of the immune-suppressive tumor microenvironment. CD4+FOXP3+Tregs are a subset of T cells with immunosuppressive properties. As shown in Figs. 7F and G, there was a significant reduction in the proportions of Tregs in the CD4+T cells and MDSCs in the CD45+cells in the treatment groups. The percentages of Tregs and MDSCs were significantly decreased compared with the control group: JK184 (p < 0.01 and p < 0.01, respectively), PD1 (p < 0.01 and p < 0.01, respectively), and JK184+PD1 (p < 0.0001 and p < 0.0001, respectively). Furthermore, JK184+PD1 regulated the polarization of macrophages. Although there was no significant difference in the proportions of the total macrophages between the treatment group and control group, the ratio of M1-like macrophages (M1) to M2-like macrophages (M2) increased compared with the control group in the JK184 (p < 0.01), PD1 (p < 0.01), and JK184+PD1 (p < 0.0001) groups (Additional file 1: Fig. S5). Our results demonstrated that JK184 could promote T cell infiltration, reshape the TME, and combine with PD1 to enhance the antitumor immune response.

## Discussion

Here, through screening compounds library, our study found three drugs (BML284/PPP/JK184) that might have a role in promoting T cell killing of tumor cells. BML284 has been proved to induce overexpressing β-catenin in response to Wnt signaling activity [31], however, recent study showed the administration of BML284 generated tumor-suppressive secretome reduced tumor growth [36]. Picropodophyllin (PPP) is a selective IGF-1R inhibitor and is involved in inhibiting tumor growth [32, 37]. In this study, we focused on JK184, which was initially exploited as a Hedgehog signaling pathway inhibitor, have recently been discovered to be a potent inhibitor of BC and CRC cells and a potential adjuvant for B7-H3 CAR T cell therapy. JK184 induces BC and CRC cell death through Hedgehog pathway inhibition, and turns B7-H3 CAR T cells into effector memory cells.

In recent years, ICB and CAR T cell therapy have played an important role in the treatment of cancer. These immunotherapy agents have shifted the paradigm of cancer treatment by increasing the responses of T cells or arming T cells with various CARs. However, despite encouraging developments, limited success has been obtained from these immunotherapy agents due to tumor heterogeneity and complicated microenvironment. Small molecule inhibitors have become a potential combination strategy to pair immunotherapies, which have greater exposure in the tumor microenvironment and access to intracellular targets. As reported in previous studies, mitogen-activated protein kinase kinase (MEK) and cyclin-dependent kinases 4 and 6 (CDK4/6) inhibitors could directly alter immune cell function and contribute to antitumor immunity synergized with PD-L1 blockade [13, 14]. BRD4, a member of the bromodomain and extraterminal (BET) subfamily of human bromodomain proteins, whose inhibitor combined with EGFR CAR-T cells suppressed the growth and metastasis of glioblastoma cells and prolonged survival in mice [28]. Another study provided evidence that IGF1R/IR inhibitors could be emerging as an adjuvant of GD2 CAR T cells therapy for diffuse midline gliomas [26]. Therefore, combining small molecule inhibitors, which induce dramatic tumor regression or reprogramming tumor microenvironment, with immunotherapies, which cannot be promoted, is an attractive potential treatment strategy.

T cells engineered with chimeric antigen receptors (CARs) can recognize tumor antigens specifically, trigger T cell activation in a non-major histocompatibility complex (MHC)-restricted manner, and initiate an extraordinary anti-tumor response [38]. In 2017, FDA approved CD19-directed CAR-T cells as a treatment for relapsed/refractory pediatric and young-adult diffuse large B cell lymphoma (DLBCL), indicating the success in monoclonal diseases and neoplastic cells with the same target antigen [16, 39]. However, while its success is well acknowledged in certain hematological malignancies, the treatment effect in solid tumors is not satisfactory, such as limited persistence and poor tumor infiltration. Specifically, the immunosuppressive tumor microenvironment (TME) interferes with CAR T cell activity in terms of differentiation and exhaustion. In addition to breaking the TME and improving the trafficking efficacy of CAR T cells to tumor sites, obstacles to using rational target antigens and preventing potential “on-target/off-tumor” toxicities remain to be overcome for CAR T cells against solid cancers. Studies have shown that epidermal growth factor receptors (EGFR, HER2), carcinoembryonic antigen (CEA) and GD2 are targets for CAR T cells against numerous solid tumors. However, a variety of reasons, including heterogeneous antigen expression or antigen expression in normal tissues, have caused failures in CAR T clinical trials [40–42]. As reported, B7-H3 has shown wide overexpression in human malignancies but limited expression in normal tissues, which has emerged as an attractive target for immunotherapies in solid cancers, including serving as a target for monoclonal antibodies, bispecific antibodies and CAR T cells. From previous studies, B7-H3 CAR T cells displayed significant antitumor activity against many solid cancers and xenografts, and have shown a certain efficacy in clinical treatment (ClinicalTrials.gov: NCT04385173, NCT05211557, NCT05241392) [43–45]. Similarly, B7-H3 CAR T cells generated in this study exhibited good effects in a two-dimensional (2D) tumor cell coculture model, three-dimensional (3D) spheroid models and mouse models. Importantly, we found that JK184 enhanced B7-H3 CAR T cells activity in the CD45RO-CD62L labeling flow cytometry assay, and observed that JK184 increased the trafficking efficacy of CAR T cells from the IHC assay.

The Hedgehog pathway is normally in charge of regulating cell growth and proliferation and consists of PTCH1, SMO, and GLI transcription factors. However, the Hedgehog pathway is aberrantly activated in multiple cancer types, including basal cell carcinoma, medulloblastoma and breast cancer, and is related to tumor invasion, metastasis, and multidrug resistance [46–48]. Therefore, Hedgehog signaling pathway inhibitors have been developed for the treatment of cancer, such as Vismodegib and Sonidegib. Both are the hedgehog pathway inhibitors that have been approved by the Food and Drug Administration (FDA) for the therapy of basal cell carcinomas, and side effects are mildly observed, but resistance is not bypassed in the treatment cases [49, 50]. It is worth noting that both Vismodegib and Sonidegib inhibit tumor growth by binding to the SMO receptor; thus, cancer cells with mutations downstream of SMO are resistant to these antagonists. In our study, JK184 was screened from compounds that inhibited downstream targets (GLI) of SMO in the Hedgehog signaling pathway and displayed good antitumor effects in vitro and in vivo. The Hedgehog signaling pathway was blocked by the binding of JK184 to GLI transcription factors, relieving GLI’s inhibition of apoptosis induced by BCL-2, which was proven by RNA sequencing and apoptotic analysis. Based on mRNA profiling, a volcano plot showed that the JUN, GZMB, IFNG, IL12RB2 and BCL6 genes were upregulated, whereas genes such as FOXP3 were downregulated in JK184-treated T cells. Meanwhile, from GSEA and the signaling pathway enrichment results, the MAPK signaling pathway was enriched, but Hedgehog pathway enrichment was not observed. We could partially elaborate the increase in T cells cytokine secretion by upregulation of the MAPK signaling pathway and the Toll-like receptor cascade pathway, but the mechanism is not clear and additional studies are needed to elucidate this possibility.

Although the majority of antitumor studies have made marked progress by using the transplantation of human cancer cells into immunodeficient mice to evaluate human CAR T cell persistence, homing, tumor control, and survival following treatment, immunodeficient mice cannot mimic the interplay of the endogenous immune system. To address this issue, we also used an immunocompetent mouse model to test the antitumor activity of JK184. To date, few reports have investigated the effects of Hedgehog inhibitors on immune cells or their potential to be combined with ICB-based immunotherapy. Of mention, the relevance of Hedgehog signaling in controlling a complex metabolic network in immune cells was highlighted in a recent study [35]. Also, Petty et al. reported that Hedgehog signaling pathway promoted tumor-associated macrophage polarization to suppress intratumoral CD8+T cell recruitment [51, 52]. Meanwhile, Mehlman C et al. have highlighted that Hedgehog pathway activation in human tumors is associate with ICB resistance [53, 54]. Furthermore, Hedgehog pathway inhibition significantly upregulated the infiltration of cytotoxic CD8 T-cells and reversed the immune suppressive microenvironment in mice models [54–56]. In the current study, we found that JK184 and ICB combinatorial treatment prolonged the survival of mice and induced CRC and BC regression in immunocompetent murine models. Here, we propose that the combination treatment synergistically increased antitumor activity through at least two parallel mechanisms. First, JK184, as a small molecule, could easily access cancer settings and penetrate into tumor cells to induce cancer cell apoptosis directly. Second, JK184 could be an adjuvant when combined with ICB treatment, which is able to promote T cell infiltration to further activate the immune system and reshape the TME. From the analysis of the tumor immune microenvironment, effector CD8^+^ T cells increased, and immunosuppressive cells, including FOXP3^+^ regulatory T cells (Tregs), myeloid-derived suppressor cells (MDSCs) and CD206^+^ macrophages (M2), decreased. Meanwhile, IFN-γ and granzyme B were detected, which are secreted by tumor-infiltrating T cells and play an important role in tumor inhibition. Our data indicated that JK184 has multifaceted antitumor effects in this JK184 plus ICB combination setting, which not only induces tumor cell death but also activates the immune response.

In conclusion, we generated second-generation B7-H3 CAR T cells, screened several compounds, displayed JK184 antitumor efficiency, showed JK184-mediated effector memory differentiation of B7-H3 CAR T cells, and provided evidence of increased cytokine secretion by JK184-treated T cells. The JK184 and B7-H3 CAR T cell combination displayed increased antitumor activity in xenograft models. Moreover, JK184 combined with ICB exhibited similarly increased antitumor activity by transforming the TME in murine models. Here we present a new combined therapeutic avenue for immunotherapy, which may enlighten future preclinical cancer research.

## Supplementary Information

**Additional file 1: Fig. S1.** Production of B7-H3 CAR T Cells. (A) Schematic representation of the B7-H3 CAR vector including a J42-scFv, linker, hinge, CD8 transmembrane domain, intracellular signaling domain of 4-1BB, and CD3-z, P2A, and mCherry. (B and C) Representative image of B7-H3 CAR expression in human T cells, which was detected using mCherry and analyzed using flow cytometry. (D) Flow cytometry analysis of the expression of B7-H3 in MDA-MB-231 and HCT116 cells. Cells were incubated with B7-H3-PE (red) or its corresponding isotype control (blue). (E) ^51^Cr-release assay to measure the cytotoxicity of B7-H3 CAR T cells against MDA-MB-231 and HCT116 cells at different E:T ratios. All error bars represent SD. T tests were used to determine statistical significance of the differences in (E). *P < 0.05, **P < 0.01, ***P < 0.001. **Fig. S2.** BML284/PPP/JK184 inhibited tumor cells and enhanced B7-H3 CAR T cells antitumor activity. (A) IC50 of BML284/PPP/JK184 in MDA-MB-231 and HCT116 cells. (B) Cells were treated with BML284/PPP**/**JK184 (1 µM) for 24h, then flow cytometry analysis of the expression of B7-H3 in MDA-MB-231 and HCT116 cells. (C) T cells were treated with different concentrations of BML284/PPP**/**JK184. (D, E and F) The expression levels of CD25, CD69, PD1 and LAG3 were detected by FACS after T cells and B7-H3 CAR T cells were treated with BML284/PPP**/**JK184 (1 µM) for 24h. T tests were used to determine statistical significance of the differences in (F). **P < 0.01, ***P < 0.001, ns not significant. (G) Diagram showing the residual tumor cells estimated from the crystal violet staining after the tumor cells were inhibited by CAR T cells or CAR T cells combined with BML284/PPP/JK184. **Fig. S3.** Identification of JK184 as a Hedgehog inhibitor. (A) KEGG analysis highlighted the breast cancer, Hedgehog signaling pathway, and apoptosis alternations in JK184-treated MDA-MB-231 cells versus nontreated MDA-MB-231 cells (WT). Each group comprised three replicates. (B) Heatmap showing the expression of hedgehog signaling target-related genes (fold change) in WT and JK184-treated MDA-MB-231 cells. (C) Real-time PCR results confirming the regulation of hedgehog signaling by the target genes SMO, PTCH1, GLI1, GLI2, GLI3 and SUFU. Each sample group comprised three replicates. **Fig. S4.** Mouse body weight was calculated in multiple mouse models. Body weight for each mouse was evaluated every 3 days in the MDA-MB-231 tumor-bearing mouse model (A), HCT116 tumor-bearing mouse model (B), 4T1 tumor-bearing mouse model (C), MC38 tumor-bearing mouse model (PD1 combined with JK184) (D) and MC38 tumor-bearing mouse model (CTLA4 combined with JK184) (E). **Fig. S5.** JK184 skews macrophages to an M1-like phenotype in the tumor microenvironment. Flow cytometry assays to analyze the macrophages of the TME in PD1 combined with JK184-treated MC38 tumors. CD11b^+^ CD86^+^ macrophagess and CD11b^+^ CD206^+^ macrophages represent M1-like macrophages (M1) and M2-like macrophages (M2) respectivelay. Representative images of (A) M1, (B) M2 and (C) the ratio of M1/M2. T tests were used to determine statistical significance of the differences in (C). **P < 0.01, ****P < 0.0001, ns not significant. **Table S1.** The antibodies for flow cytometry. **Table S2.** Primer nucleotide sequences for qRT-PCR.

## Data Availability

Data are available in a public, open access repository. The RNA-seq datasets of MDA-MB-231 cells and T cells generated during this study are available at SRA: PRJNA818783 and SRA: PRJNA819165, respectively. Further information and requests for resources and reagents should be directed to and will be fulfilled by the corresponding author.
