# Supplementary material for: A drug screening to identify novel combinatorial strategies for boosting cancer immunotherapy efficacy
Source: J Transl Med. 2023 Jan 13;21:23. doi: 10.1186/s12967-023-03875-4 (PMC9838049; doi:10.1186/s12967-023-03875-4)
Supplement: Supplementary file 1 — Additional file 1: Fig. S1. Production of B7-H3 CAR T Cells. (A) Schematic representation of the B7-H3 CAR vector including a J42-scFv, linker, hinge, CD8 transmembrane domain, intracellular signaling domain of 4-1BB, and CD3-z, P2A, and mCherry. (B and C) Representative image of B7-H3 CAR expression in human T cells, which was detected using mCherry and analyzed using flow cytometry. (D) Flow cytometry analysis of the expression of B7-H3 in MDA-MB-231 and HCT116 cells. Cells were incubated with B7-H3-PE (red) or its corresponding isotype control (blue). (E) 51Cr-release assay to measure the cytotoxicity of B7-H3 CAR T cells against MDA-MB-231 and HCT116 cells at different E:T ratios. All error bars represent SD. T tests were used to determine statistical significance of the differences in (E). *P < 0.05, **P < 0.01, ***P < 0.001. Fig. S2. BML284/PPP/JK184 inhibited tumor cells and enhanced B7-H3 CAR T cells antitumor activity. (A) IC50 of BML284/PPP/JK184 in MDA-MB-231 and HCT116 cells. (B) Cells were treated with BML284/PPP/JK184 (1 µM) for 24h, then flow cytometry analysis of the expression of B7-H3 in MDA-MB-231 and HCT116 cells. (C) T cells were treated with different concentrations of BML284/PPP/JK184. (D, E and F) The expression levels of CD25, CD69, PD1 and LAG3 were detected by FACS after T cells and B7-H3 CAR T cells were treated with BML284/PPP/JK184 (1 µM) for 24h. T tests were used to determine statistical significance of the differences in (F). **P < 0.01, ***P < 0.001, ns not significant. (G) Diagram showing the residual tumor cells estimated from the crystal violet staining after the tumor cells were inhibited by CAR T cells or CAR T cells combined with BML284/PPP/JK184. Fig. S3. Identification of JK184 as a Hedgehog inhibitor. (A) KEGG analysis highlighted the breast cancer, Hedgehog signaling pathway, and apoptosis alternations in JK184-treated MDA-MB-231 cells versus nontreated MDA-MB-231 cells (WT). Each group comprised three replicates. (B) Heat [file 12967_2023_3875_MOESM1_ESM.docx]

**Additional file1: Fig.S1**
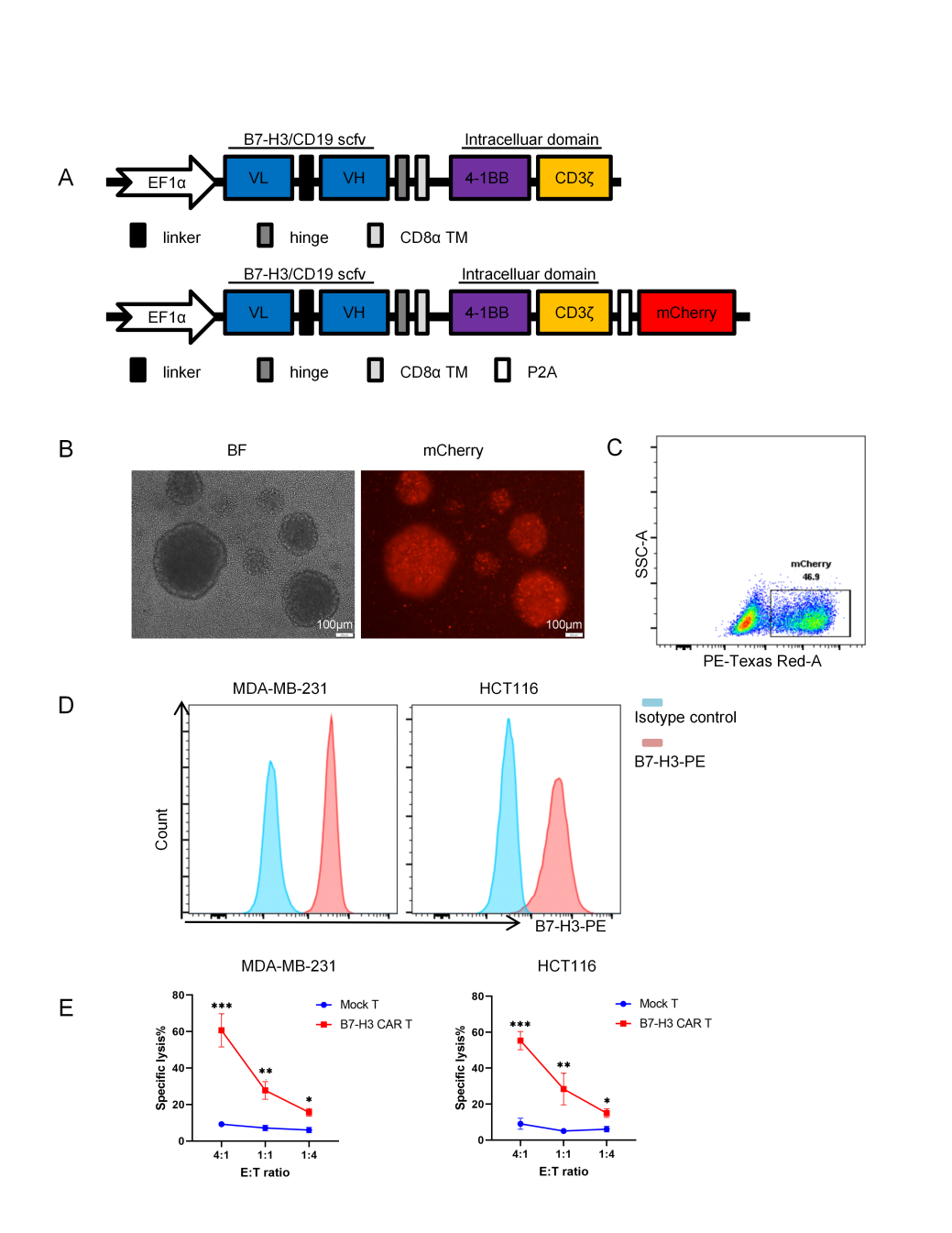


**Production of B7-H3 CAR T Cells.** (A) Schematic representation of the B7-H3 CAR vector including a J42-scFv, linker, hinge, CD8 transmembrane domain, intracellular signaling domain of 4-1BB, and CD3-z, P2A, and mCherry. (B and C) Representative image of B7-H3 CAR expression in human T cells, which was detected using mCherry and analyzed using flow cytometry. (D) Flow cytometry analysis of the expression of B7-H3 in MDA-MB-231 and HCT116 cells. Cells were incubated with B7-H3-PE (red) or its corresponding isotype control (blue). (E) ^51^Cr-release assay to measure the cytotoxicity of B7-H3 CAR T cells against MDA-MB-231 and HCT116 cells at different E:T ratios. All error bars represent SD. T tests were used to determine statistical significance of the differences in (E). *P < 0.05, **P < 0.01, ***P < 0.001.

**Additional file 1: Fig.S2**


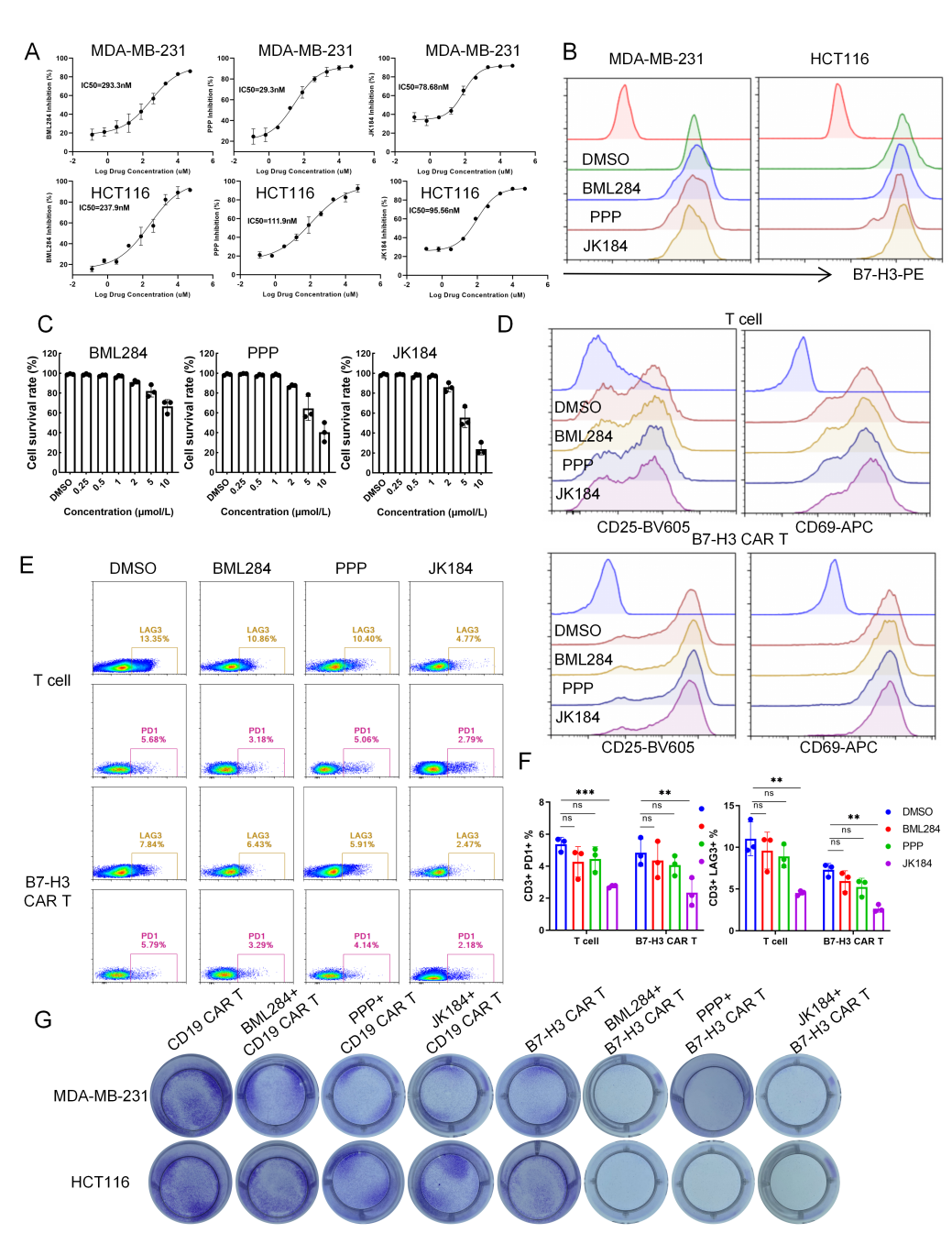


**BML284/PPP/JK184 inhibited tumor cells and enhanced B7-H3 CAR T cells antitumor activity.** (A) IC50 of BML284/PPP**/**JK184 in MDA-MB-231 and HCT116 cells. (B) Cells were treated with BML284/PPP**/**JK184 (1 µM) for 24h, then flow cytometry analysis of the expression of B7-H3 in MDA-MB-231 and HCT116 cells. (C) T cells were treated with different concentrations of BML284/PPP**/**JK184. (D, E and F) The expression levels of CD25, CD69, PD1 and LAG3 were detected by FACS after T cells and B7-H3 CAR T cells were treated with BML284/PPP**/**JK184 (1 µM) for 24h. T tests were used to determine statistical significance of the differences in (F). **P < 0.01, ***P < 0.001, ns not significant. (G) Diagram showing the residual tumor cells estimated from the crystal violet staining after the tumor cells were inhibited by CAR T cells or CAR T cells combined with BML284/PPP/JK184.

**Additional file 1 Fig.S3**


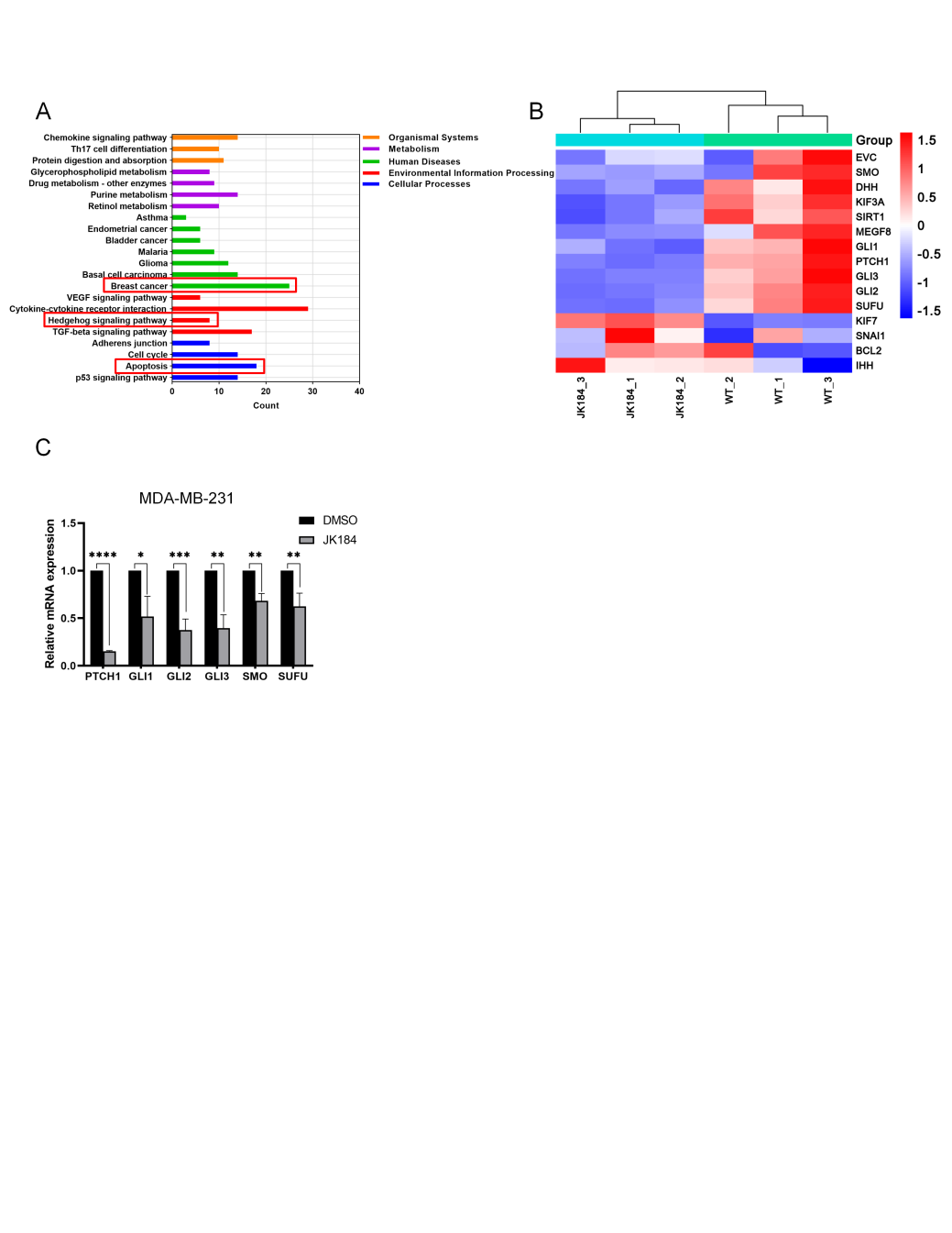


**Identification of JK184 as a Hedgehog inhibitor.** (A) KEGG analysis highlighted the breast cancer, Hedgehog signaling pathway, and apoptosis alternations in JK184-treated MDA-MB-231 cells versus nontreated MDA-MB-231 cells (WT). Each group comprised three replicates. (B) Heatmap showing the expression of hedgehog signaling target-related genes (fold change) in WT and JK184-treated MDA-MB-231 cells. (C) Real-time PCR results confirming the regulation of hedgehog signaling by the target genes SMO, PTCH1, GLI1, GLI2, GLI3 and SUFU. Each sample group comprised three replicates.

**Additional file 1 Fig.S4**


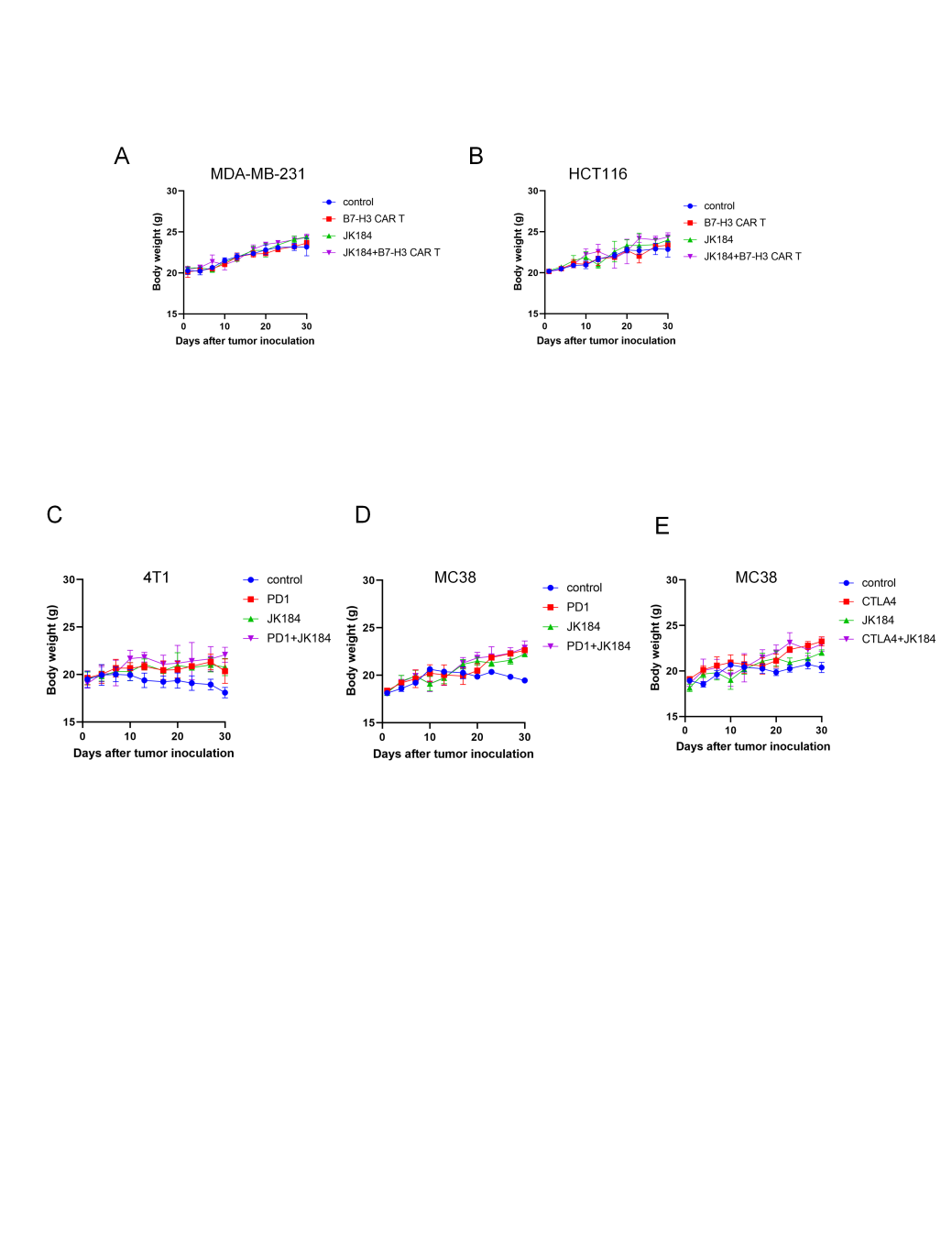


**Mouse body weight was calculated in multiple mouse models.** Body weight for each mouse was evaluated every three days in the MDA-MB-231 tumor-bearing mouse model (A), HCT116 tumor-bearing mouse model (B), 4T1 tumor-bearing mouse model (C), MC38 tumor-bearing mouse model (PD1 combined with JK184) (D) and MC38 tumor-bearing mouse model (CTLA4 combined with JK184) (E).

**Additional file 1 Fig.S5**


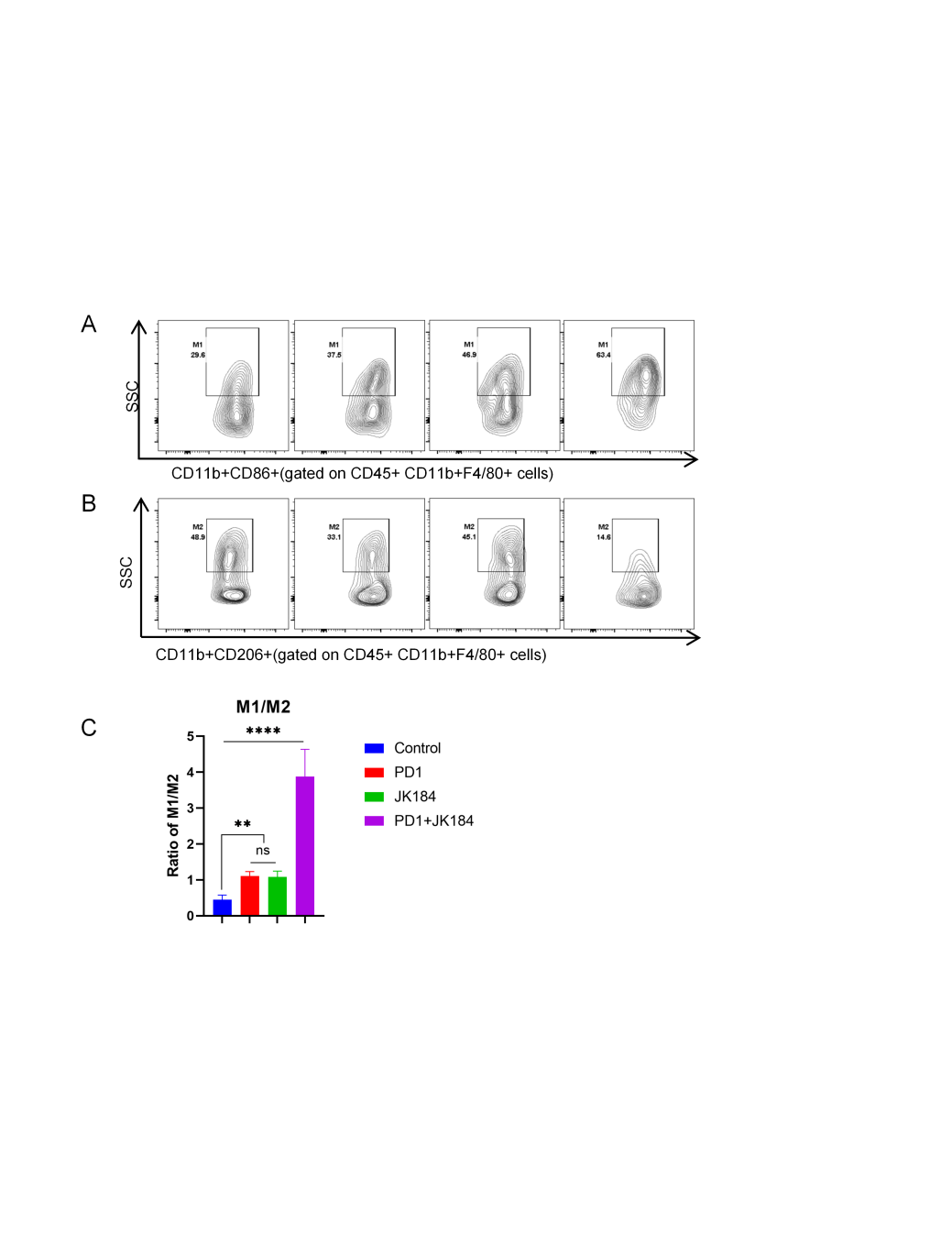


**JK184 skews macrophages to an M1-like phenotype in the tumor microenvironment.** Flow cytometry assays to analyze the macrophages of the TME in PD1 combined with JK184-treated MC38 tumors. CD11b^+^ CD86^+^ macrophagess and CD11b^+^ CD206^+^ macrophages represent M1-like macrophages (M1) and M2-like macrophages (M2) respectivelay. Representative images of (A) M1, (B) M2 and (C) the ratio of M1/M2. T tests were used to determine statistical significance of the differences in (C). **P < 0.01, ****P < 0.0001, ns not significant.

**Additional file 1 Table S1. The antibodies for flow cytometry**

| Antibodies | SOURCE | IDENTIFIER |
| --- | --- | --- |
| Anti-human PE-CY7-CD45RA | BioLegend | Cat# 304126 |
| Anti-human FITC-CD62L | BioLegend | Cat# 304804 |
| Anti-human Percp-CD3 | BioLegend | Cat# 300428 |
| Anti-human APC-Perforin | BioLegend | Cat# 308111 |
| Anti-human/mouse APC-Granzyme B | BioLegend | Cat# 372204 |
| Trustain fcX anti-mouse CD16/CD32 | BioLegend | Cat# 40477 |
| Anti-mouse APC-CY7-CD45 | BD | Cat# 561037 |
| Anti-mouse Percp-CD45 | BioLegend | Cat# 103132 |
| Anti-mouse APC-CY7-CD3 | BD | Cat# 560590 |
| Anti-mouse Percp-CD3 | BioLegend | Cat# 100218 |
| Anti-mouse FITC-CD4 | BD | Cat# 553650 |
| Anti-mouse BV510-CD8a | BioLegend | Cat# 100752 |
| Anti-mouse FITC-CD11b | BioLegend | Cat# 101206 |
| Anti-mouse PE-F4/80 | BioLegend | Cat# 123110 |
| Anti-mouse PE-CY7-CD86 | BD | Cat# 560582 |
| Anti-mouse APC-CD206 | BD | Cat# 565250 |
| Anti-mouse BV510-Gr-1 | BioLegend | Cat# 108438 |
| Anti-mouse PE-FOXP3 | BioLegend | Cat# 126404 |
| Anti-mouse BV421-IFN-γ | BioLegend | Cat# 505830 |

**Additional file 1 Table S2. Primer nucleotide sequences for qRT-PCR**

| Gene | Forward primer (5′-3′) | Reverse primer (5′-3′) |
| --- | --- | --- |
| Ptch1 | AATCAGGGGAACTTATCA | ACCGTAAAGGAGGCTTA |
| Gli-1 | GGATACAACCCAAATGC | TGGCGAATAGACAGAGG |
| Gli-2 | TGACCGAAGTGACGATG | AAGTATGGGGAGATGCC |
| Gli-3 | CCCCTACATCAACCCAT | CCTGTCAGCAGAGCCAT |
| Smo | CTGACTTTCTGCGTTGC | TTGGGGTTGTCTGTTCG |
| SUFU | ACACCCGTATGCAGCTCA | GTCGGGGAGAATGGACACT |
| GAPDH | CCTTCCGTGTTCCTAC | GACAACCTGGTCCTCA |
| BCL6 | CTGCAGATGGAGCATGTTGT | GCCATTTCTGCTTCACTGG |
| FOXP3 | GGTATTGAGGGTGGGTGTCA | AGCTGCTGAGATGTGACTGT |
| GZMB | AGGTGCGGTGGCTTCCTGATAC | CTGGGTCGGCTCCTGTTCTTTG |
| IL12RB2 | AGACCTCAGTGGTGTAGCAGAG | TGATGACCAGCGGTTCAGGATC |
| IFNG | ACGGCACAGTCATTGAAAGC | TGCTGATGGCCTGATTGTCTT |
| JUN | CCTTGAAAGCTCAGAACTCGGAG | TGCTGCGTTAGCATGAGTTGGC |
